# Supplementary material for: First Organic–Inorganic Hybrid Compounds Formed by Ge-V-O Clusters and Transition Metal Complexes of Aromatic Organic Ligands
Source: Molecules. 2022 Jul 11;27(14):4424. doi: 10.3390/molecules27144424 (PMC9323094; doi:10.3390/molecules27144424)

checkCIF/PLATON report

You have not supplied any structure factors. As a result the full set of tests cannot be run.

THIS REPORT IS FOR GUIDANCE ONLY. IF USED AS PART OF A REVIEW PROCEDURE FOR PUBLICATION, IT SHOULD NOT REPLACE THE EXPERTISE OF AN EXPERIENCED CRYSTALLOGRAPHIC REFEREE.

No syntax errors found. CIF dictionary Interpreting this report

Datablock: k1

Bond precision: C-C = 0.0148 A Wavelength=0.71073

Cell:	a=14.5034(8)	b=16.5920(9)	c=23.0440(13)
	alpha=71.648(4)	beta=84.130(4)	gamma=75.454(4)
Temperature:	293 K		

	Calculated	Reported
Volume	5093.0(5)	5093.0(5)
Space group	P -1	P -1
Hall group	-P 1	-P 1
Moiety formula	C104 H96 Cd8 Ge16 N24 O98 V24, 25(O)	C52 H48 Cd4 Ge8 N12 O49 V12, 12.5(O)
Sum formula	C104 H96 Cd8 Ge16 N24 O123 V24	C52 H83 Cd4 Ge8 N12 O61.50 V12
Mr	6933.65	3501.90
Dx,g cm-3	2.261	2.284
Z	1	2
Mu (mm-1)	4.281	4.282
F000	3320.0	3390.0
F000'	3324.66	
h,k,lmax	17,19,27	17,19,27
Nref	18009	17941
Tmin,Tmax	0.422,0.549	0.607,0.612
Tmin'	0.386	

Correction method= # Reported T Limits: Tmin=0.607 Tmax=0.612
AbsCorr = MULTI-SCAN

Data completeness= 0.996 Theta(max)= 25.032

R(reflections)= 0.0621(14416)

wR2(reflections)= 0.1660(17941)

S = 1.030 Npar= 1360

The following ALERTS were generated. Each ALERT has the format
test-name_ALERT_alert-type_alert-level.
Click on the hyperlinks for more details of the test.

Alert level B

PLAT043_ALERT_1_B Calculated and Reported Mol. Weight Differ by .. 70.15 Check

Author Response: The hydrogens of all the water molecules was not added.

PLAT306_ALERT_2_B Isolated Oxygen Atom (H-atoms Missing ?) .... O1W Check

Author Response: The hydrogens of the water molecule was not added.

PLAT306_ALERT_2_B Isolated Oxygen Atom (H-atoms Missing ?) .... O2W Check

Author Response: The hydrogens of the water molecule was not added.

PLAT306_ALERT_2_B Isolated Oxygen Atom (H-atoms Missing ?) .... O3W Check

Author Response: The hydrogens of the water molecule was not added.

PLAT306_ALERT_2_B Isolated Oxygen Atom (H-atoms Missing ?) .... O4W Check

Author Response: The hydrogens of the water molecule was not added.

PLAT306_ALERT_2_B Isolated Oxygen Atom (H-atoms Missing ?) .... O5W Check

Author Response: The hydrogens of the water molecule was not added.

PLAT306_ALERT_2_B Isolated Oxygen Atom (H-atoms Missing ?) .... O6W Check

Author Response: The hydrogens of the water molecule was not added.

PLAT306_ALERT_2_B Isolated Oxygen Atom (H-atoms Missing ?) .... O7W Check

Author Response: The hydrogens of the water molecule was not added.

```
PLAT306_ALERT_2_B Isolated Oxygen Atom (H-atoms Missing ?) .......        O8W Check
```

Author Response: The hydrogens of the water molecule was not added.

```
PLAT306_ALERT_2_B Isolated Oxygen Atom (H-atoms Missing ?) .......       O10W Check
```

Author Response: The hydrogens of the water molecule was not added.

```
PLAT306_ALERT_2_B Isolated Oxygen Atom (H-atoms Missing ?) .......       O12W Check
```

Author Response: The hydrogens of the water molecule was not added.

```
PLAT306_ALERT_2_B Isolated Oxygen Atom (H-atoms Missing ?) .......       O13W Check
```

Author Response: The hydrogens of the water molecule was not added.

```
PLAT430_ALERT_2_B Short Inter D...A Contact  O1       ..O0AA     .       2.59 Ang.
                                                   x,y,z  =      1_555 Check
```

Author Response: There should be a strong hydrogen bond between the two atoms.

```
PLAT430_ALERT_2_B Short Inter D...A Contact  O1       ..O4W      .       2.65 Ang.
                                                   x,y,z  =      1_555 Check
```

Author Response: There should be a strong hydrogen bond between the two atoms.

```
PLAT430_ALERT_2_B Short Inter D...A Contact  O2W      ..O6       .       2.80 Ang.
                                              2-x,-y,1-z  =      2_756 Check
```

Author Response: There should be a strong hydrogen bond between the two atoms.

```
PLAT430_ALERT_2_B Short Inter D...A Contact  O2W      ..O7       .       2.83 Ang.
                                              2-x,-y,1-z  =      2_756 Check
```

Author Response: There should be a strong hydrogen bond between the two atoms.

```
PLAT430_ALERT_2_B Short Inter D...A Contact  O3       ..O13W     .       2.78 Ang.
                                                   x,y,z  =      1_555 Check
```

Author Response: There should be a strong hydrogen bond between the two atoms.

```
PLAT430_ALERT_2_B Short Inter D...A Contact  O3W      ..O4W      .       2.76 Ang.
                                             1-x,1-y,1-z  =      2_666 Check
```

Author Response: There should be a strong hydrogen bond between the two atoms.

```
PLAT430_ALERT_2_B Short Inter D...A Contact  O4W       ..O5W      .       2.84 Ang.
                                                -1+x,y,z  =      1_455 Check
```

Author Response: There should be a strong hydrogen bond between the two atoms.

```
PLAT430_ALERT_2_B Short Inter D...A Contact  O6        ..O6W      .       2.77 Ang.
                                                   x,y,z  =      1_555 Check
```

Author Response: There should be a strong hydrogen bond between the two atoms.

```
PLAT430_ALERT_2_B Short Inter D...A Contact  O6W       ..O13W     .       2.73 Ang.
                                                 1+x,y,z  =      1_655 Check
```

Author Response: There should be a strong hydrogen bond between the two atoms.

```
PLAT430_ALERT_2_B Short Inter D...A Contact  O7W       ..O10W     .       2.76 Ang.
                                             1-x,1-y,1-z  =      2_666 Check
```

Author Response: There should be a strong hydrogen bond between the two atoms.

```
PLAT430_ALERT_2_B Short Inter D...A Contact  O8        ..O24      .       2.78 Ang.
                                              1-x,1-y,-z  =      2_665 Check
```

Author Response: There should be a strong hydrogen bond between the two atoms.

```
PLAT430_ALERT_2_B Short Inter D...A Contact  O8W       ..O15      .       2.79 Ang.
                                                   x,y,z  =      1_555 Check
```

Author Response: There should be a strong hydrogen bond between the two atoms.

```
PLAT430_ALERT_2_B Short Inter D...A Contact  O10       ..O32      .       2.76 Ang.
                                              2-x,1-y,-z  =      2_765 Check
```

Author Response: There should be a strong hydrogen bond between the two atoms.

```
PLAT430_ALERT_2_B Short Inter D...A Contact  O10W      ..O14      .       2.70 Ang.
                                                   x,y,z  =      1_555 Check
```

Author Response: There should be a strong hydrogen bond between the two atoms.

```
PLAT430_ALERT_2_B Short Inter D...A Contact  O11W     ..O13      .       2.73 Ang.
                                                       x,y,z  =      1_555 Check
```

Author Response: There should be a strong hydrogen bond between the two atoms.

```
PLAT430_ALERT_2_B Short Inter D...A Contact  O12W     ..O21      .       2.80 Ang.
                                                       x,y,z  =      1_555 Check
```

Author Response: There should be a strong hydrogen bond between the two atoms.

```
PLAT430_ALERT_2_B Short Inter D...A Contact  O14W     ..O44      .       2.83 Ang.
                                                       x,y,z  =      1_555 Check
```

Author Response: There should be a strong hydrogen bond between the two atoms.

Alert level C

```
RINTA01_ALERT_3_C  The value of Rint is greater than 0.12
            Rint given   0.126
PLAT041_ALERT_1_C Calc. and Reported SumFormula    Strings  Differ     Please Check
PLAT068_ALERT_1_C Reported F000 Differs from Calcd (or Missing)...     Please Check
PLAT220_ALERT_2_C NonSolvent   Resd 1  C   Ueq(max)/Ueq(min) Range        4.0 Ratio
PLAT220_ALERT_2_C NonSolvent   Resd 1  O   Ueq(max)/Ueq(min) Range        4.2 Ratio
PLAT234_ALERT_4_C Large Hirshfeld Difference C5       --C6       .       0.18 Ang.
PLAT241_ALERT_2_C High   'MainMol' Ueq as Compared to Neighbors of         C2 Check
PLAT241_ALERT_2_C High   'MainMol' Ueq as Compared to Neighbors of        C49 Check
PLAT241_ALERT_2_C High   'MainMol' Ueq as Compared to Neighbors of        C50 Check
PLAT242_ALERT_2_C Low    'MainMol' Ueq as Compared to Neighbors of         N9 Check
PLAT242_ALERT_2_C Low    'MainMol' Ueq as Compared to Neighbors of        N10 Check
PLAT260_ALERT_2_C Large Average Ueq of Residue Including      O0AA      0.146 Check
PLAT260_ALERT_2_C Large Average Ueq of Residue Including       O1W      0.262 Check
PLAT260_ALERT_2_C Large Average Ueq of Residue Including       O2W      0.231 Check
PLAT260_ALERT_2_C Large Average Ueq of Residue Including       O3W      0.169 Check
PLAT260_ALERT_2_C Large Average Ueq of Residue Including       O4W      0.161 Check
PLAT260_ALERT_2_C Large Average Ueq of Residue Including       O5W      0.153 Check
PLAT260_ALERT_2_C Large Average Ueq of Residue Including       O6W      0.114 Check
PLAT260_ALERT_2_C Large Average Ueq of Residue Including      O12W      0.121 Check
PLAT260_ALERT_2_C Large Average Ueq of Residue Including      O13W      0.139 Check
PLAT342_ALERT_3_C Low Bond Precision on  C-C Bonds ...............    0.01476 Ang.
PLAT360_ALERT_2_C Short  C(sp3)-C(sp3) Bond  C49      - C50      .       1.38 Ang.
PLAT420_ALERT_2_C D-H Bond Without Acceptor  N9       --H9A      .     Please Check
PLAT430_ALERT_2_C Short Inter D...A Contact  O2W      ..O3W      .       2.86 Ang.
                                                       x,y,z  =      1_555 Check
```

Author Response: There should be a strong hydrogen bond between the two atoms.

```
PLAT430_ALERT_2_C Short Inter D...A Contact  O7W      ..O19      .       2.89 Ang.
                                                       x,y,z  =      1_555 Check
```

Author Response: There should be a strong hydrogen bond between the two atoms.

```
PLAT430_ALERT_2_C Short Inter D...A Contact  O9W      ..O32      .       2.85 Ang.
                                               2-x,1-y,-z  =      2_765 Check
```

Author Response: There should be a strong hydrogen bond between the two atoms.

```
PLAT430_ALERT_2_C Short Inter D...A Contact  O10W     ..O11      .       2.86 Ang.
                                              2-x,1-y,1-z  =      2_766 Check
```

Author Response: There should be a strong hydrogen bond between the two atoms.

```
PLAT430_ALERT_2_C Short Inter D...A Contact  O13      ..O14W     .       2.89 Ang.
                                                    x,y,z  =      1_555 Check
```

Author Response: There should be a strong hydrogen bond between the two atoms.

```
PLAT601_ALERT_2_C Unit Cell Contains Solvent Accessible VOIDS of .         32 Ang**3
```

Alert level G

```
FORMU01_ALERT_1_G  There is a discrepancy between the atom counts in the
            _chemical_formula_sum and _chemical_formula_moiety. This is
            usually due to the moiety formula being in the wrong format.
            Atom count from _chemical_formula_sum:   C52 H83 Cd4 Ge8 N12 O61.5 V12
            Atom count from _chemical_formula_moiety:C52 H48 Cd4 Ge8 N12 O61.5 V12
FORMU01_ALERT_2_G  There is a discrepancy between the atom counts in the
            _chemical_formula_sum and the formula from the _atom_site* data.
            Atom count from _chemical_formula_sum:C52 H83 Cd4 Ge8 N12 O61.5 V12
            Atom count from the _atom_site data:  C52 H48 Cd4 Ge8 N12 O61.5 V12
CELLZ01_ALERT_1_G Difference between formula and atom_site contents detected.
CELLZ01_ALERT_1_G WARNING: H atoms missing from atom site list. Is this intentional?
           From the CIF: _cell_formula_units_Z    2
           From the CIF: _chemical_formula_sum  C52 H83 Cd4 Ge8 N12 O61.50 V12
           TEST: Compare cell contents of formula and atom_site data
```

atom	Z*formula	cif sites	diff
C	104.00	104.00	0.00
H	166.00	96.00	70.00
Cd	8.00	8.00	0.00
Ge	16.00	16.00	0.00
N	24.00	24.00	0.00
O	123.00	123.00	0.00
V	24.00	24.00	0.00

```
PLAT002_ALERT_2_G Number of Distance or Angle Restraints on AtSite          4 Note
PLAT007_ALERT_5_G Number of Unrefined Donor-H Atoms .............           8 Report
PLAT020_ALERT_3_G The Value of Rint is Greater Than 0.12 .........      0.126 Report
PLAT042_ALERT_1_G Calc. and Reported Moiety Formula Strings Differ     Please Check
PLAT045_ALERT_1_G Calculated and Reported Z Differ by a Factor ...       0.50 Check
PLAT072_ALERT_2_G SHELXL First  Parameter in WGHT  Unusually Large       0.10 Report
PLAT083_ALERT_2_G SHELXL Second Parameter in WGHT  Unusually Large       6.59 Why ?
PLAT154_ALERT_1_G The s.u.'s on the Cell Angles are Equal ..(Note)      0.004 Degree
```

```
PLAT172_ALERT_4_G The CIF-Embedded .res File Contains DFIX Records          2 Report
PLAT180_ALERT_4_G Check Cell Rounding: # of Values Ending with 0 =          3 Note
PLAT199_ALERT_1_G Reported _cell_measurement_temperature ..... (K)        293 Check
PLAT200_ALERT_1_G Reported   _diffrn_ambient_temperature ..... (K)        293 Check
PLAT300_ALERT_4_G Atom Site Occupancy of O0AA       Constrained at        0.5 Check
PLAT300_ALERT_4_G Atom Site Occupancy of O11W       Constrained at        0.5 Check
PLAT300_ALERT_4_G Atom Site Occupancy of O14W       Constrained at        0.5 Check
PLAT302_ALERT_4_G Anion/Solvent/Minor-Residue Disorder (Resd  2  )       100% Note
PLAT302_ALERT_4_G Anion/Solvent/Minor-Residue Disorder (Resd 12  )       100% Note
PLAT302_ALERT_4_G Anion/Solvent/Minor-Residue Disorder (Resd 15  )       100% Note
PLAT304_ALERT_4_G Non-Integer Number of Atoms in ..... (Resd  2  )       0.50 Check
PLAT304_ALERT_4_G Non-Integer Number of Atoms in ..... (Resd 12  )       0.50 Check
PLAT304_ALERT_4_G Non-Integer Number of Atoms in ..... (Resd 15  )       0.50 Check
PLAT311_ALERT_2_G Isolated Disordered Oxygen Atom (No H's ?) .....       O0AA Check
PLAT311_ALERT_2_G Isolated Disordered Oxygen Atom (No H's ?) .....       O11W Check
PLAT311_ALERT_2_G Isolated Disordered Oxygen Atom (No H's ?) .....       O14W Check
PLAT720_ALERT_4_G Number of Unusual/Non-Standard Labels ..........          1 Note
PLAT789_ALERT_4_G Atoms with Negative _atom_site_disorder_group  #          1 Check
PLAT790_ALERT_4_G Centre of Gravity not Within Unit Cell: Resd.  #          7 Note
              O
PLAT790_ALERT_4_G Centre of Gravity not Within Unit Cell: Resd.  #          8 Note
              O
PLAT794_ALERT_5_G Tentative Bond Valency for Cd1       (II)      .       2.03 Info
PLAT794_ALERT_5_G Tentative Bond Valency for Cd2       (II)      .       1.83 Info
PLAT794_ALERT_5_G Tentative Bond Valency for Cd3       (II)      .       2.03 Info
PLAT794_ALERT_5_G Tentative Bond Valency for Cd4       (II)      .       2.08 Info
PLAT794_ALERT_5_G Tentative Bond Valency for V1        (IV)      .       4.06 Info
PLAT794_ALERT_5_G Tentative Bond Valency for V2        (IV)      .       4.02 Info
PLAT794_ALERT_5_G Tentative Bond Valency for V3        (IV)      .       4.06 Info
PLAT794_ALERT_5_G Tentative Bond Valency for V4        (IV)      .       4.13 Info
PLAT794_ALERT_5_G Tentative Bond Valency for V5        (IV)      .       4.08 Info
PLAT794_ALERT_5_G Tentative Bond Valency for V6        (IV)      .       4.13 Info
PLAT794_ALERT_5_G Tentative Bond Valency for V7        (IV)      .       4.14 Info
PLAT794_ALERT_5_G Tentative Bond Valency for V8        (IV)      .       4.04 Info
PLAT794_ALERT_5_G Tentative Bond Valency for V9        (IV)      .       4.01 Info
PLAT794_ALERT_5_G Tentative Bond Valency for V10       (IV)      .       4.16 Info
PLAT794_ALERT_5_G Tentative Bond Valency for V11       (IV)      .       4.18 Info
PLAT794_ALERT_5_G Tentative Bond Valency for V12       (IV)      .       4.19 Info
PLAT860_ALERT_3_G Number of Least-Squares Restraints ............          2 Note
PLAT941_ALERT_3_G Average HKL Measurement Multiplicity ..........         1.6 Low
```

```
   0 ALERT level A = Most likely a serious problem - resolve or explain
  29 ALERT level B = A potentially serious problem, consider carefully
  29 ALERT level C = Check. Ensure it is not caused by an omission or oversight
  50 ALERT level G = General information/check it is not something unexpected

  11 ALERT type 1 CIF construction/syntax error, inconsistent or missing data
  59 ALERT type 2 Indicator that the structure model may be wrong or deficient
   5 ALERT type 3 Indicator that the structure quality may be low
  16 ALERT type 4 Improvement, methodology, query or suggestion
  17 ALERT type 5 Informative message, check
```

Datablock: k2

Bond precision: C-C = 0.0242 A Wavelength=0.71073

Cell:	a=17.193(3)	b=23.511(5)	c=26.373(5)
	alpha=90	beta=100.15(3)	gamma=90
Temperature:	293 K		

	Calculated	Reported
Volume	10494(4)	10494(4)
Space group	C 2/c	C 1 2/c 1
Hall group	-C 2yc	-C 2yc
Moiety formula	2(C36 H55 Cd4.50 Ge8 N13 O48 V12), 5(O0.50), 4(O0.50), 4(O0.50)	0.5(C72 H110 Cd9 Ge16 N26 O96 V24), 8(O)
Sum formula	C72 H110 Cd9 Ge16 N26 O112 V24	C36 H78 Cd4.50 Ge8 N13 O56 V12
Mr	6527.88	3286.91
Dx,g cm-3	2.066	2.080
Z	2	4
Mu (mm-1)	4.241	4.242
F000	6232.0	6324.0
F000'	6239.42	
h,k,lmax	22,30,34	22,30,34
Nref	12013	11814
Tmin,Tmax	0.425,0.552	0.251,1.000
Tmin'	0.358	

Correction method= # Reported T Limits: Tmin=0.251 Tmax=1.000
AbsCorr = EMPIRICAL

Data completeness= 0.983 Theta(max)= 27.466

R(reflections)= 0.0822(6598) wR2(reflections)= 0.2629(11814)

S = 1.042 Npar= 662

The following ALERTS were generated. Each ALERT has the format
test-name_ALERT_alert-type_alert-level.
Click on the hyperlinks for more details of the test.

Alert level B

PLAT342_ALERT_3_B Low Bond Precision on C-C Bonds .... 0.02421 Ang.

Author Response: Most likely resulting from poor quality diffraction data. No better quality crystal could be obtained

```
PLAT430_ALERT_2_B Short Inter D...A Contact  O2W      ..O23      .       2.83 Ang.
                                               1-x,y,1/2-z  =      2_655 Check
```

Author Response: There should be a strong hydrogen bond between the two atoms.

```
PLAT430_ALERT_2_B Short Inter D...A Contact  O3W      ..O25      .       2.84 Ang.
                                       -1/2+x,1/2-y,-1/2+z  =      8_455 Check
```

Author Response: There should be a strong hydrogen bond between the two atoms.

```
PLAT430_ALERT_2_B Short Inter D...A Contact  O5       ..O5W      .       2.80 Ang.
                                               1-x,y,1/2-z  =      2_655 Check
```

Author Response: There should be a strong hydrogen bond between the two atoms.

```
PLAT430_ALERT_2_B Short Inter D...A Contact  O5W      ..O23      .       2.77 Ang.
                                         1/2-x,1/2+y,1/2-z  =      4_555 Check
```

Author Response: There should be a strong hydrogen bond between the two atoms.

```
PLAT430_ALERT_2_B Short Inter D...A Contact  O6       ..O4W      .       2.62 Ang.
                                               1-x,y,1/2-z  =      2_655 Check
```

Author Response: There should be a strong hydrogen bond between the two atoms.

```
PLAT430_ALERT_2_B Short Inter D...A Contact  O6W      ..O22      .       2.69 Ang.
                                                     x,y,z  =      1_555 Check
```

Author Response: There should be a strong hydrogen bond between the two atoms.

```
PLAT430_ALERT_2_B Short Inter D...A Contact  O7W      ..O22      .       2.63 Ang.
                                                     x,y,z  =      1_555 Check
```

Author Response: There should be a strong hydrogen bond between the two atoms.

```
PLAT430_ALERT_2_B Short Inter D...A Contact  O8W      ..O26      .       2.77 Ang.
                                               1-x,y,1/2-z  =      2_655 Check
```

Author Response: There should be a strong hydrogen bond between the two atoms.

```
PLAT430_ALERT_2_B Short Inter D...A Contact  O9       ..O9W      .       2.83 Ang.
                                        3/2-x,-1/2+y,1/2-z  =      4_645 Check
```

Author Response: There should be a strong hydrogen bond between the two atoms.

```
PLAT430_ALERT_2_B Short Inter D...A Contact  O9W      ..O26      .       2.74 Ang.
                                           1-x,y,1/2-z  =      2_655 Check
```

Author Response: There should be a strong hydrogen bond between the two atoms.

Alert level C

```
ABSTY02_ALERT_1_C  An _exptl_absorpt_correction_type has been given without
            a literature citation. This should be contained in the
            _exptl_absorpt_process_details field.
            Absorption correction given as empirical
PLAT031_ALERT_4_C Refined Extinction Parameter Within Range ......      2.750 Sigma
PLAT041_ALERT_1_C Calc. and Reported SumFormula    Strings  Differ     Please Check
PLAT043_ALERT_1_C Calculated and Reported Mol. Weight Differ by ..      45.94 Check
PLAT068_ALERT_1_C Reported F000 Differs from Calcd (or Missing)...     Please Check
PLAT084_ALERT_3_C High wR2 Value (i.e. > 0.25) ..................       0.26 Report
PLAT094_ALERT_2_C Ratio of Maximum / Minimum Residual Density ....       2.58 Report
PLAT213_ALERT_2_C Atom C5              has ADP max/min Ratio .....        3.5 oblate
PLAT220_ALERT_2_C NonSolvent   Resd 1  C   Ueq(max)/Ueq(min) Range        4.8 Ratio
PLAT220_ALERT_2_C NonSolvent   Resd 1  N   Ueq(max)/Ueq(min) Range        3.1 Ratio
PLAT234_ALERT_4_C Large Hirshfeld Difference N1       --C1       .       0.17 Ang.
PLAT234_ALERT_4_C Large Hirshfeld Difference N4       --C14      .       0.22 Ang.
PLAT234_ALERT_4_C Large Hirshfeld Difference N4       --C15      .       0.19 Ang.
PLAT234_ALERT_4_C Large Hirshfeld Difference N5       --C16      .       0.21 Ang.
PLAT241_ALERT_2_C High   'MainMol' Ueq as Compared to Neighbors of         N3 Check
PLAT241_ALERT_2_C High   'MainMol' Ueq as Compared to Neighbors of        C14 Check
PLAT241_ALERT_2_C High   'MainMol' Ueq as Compared to Neighbors of        C15 Check
PLAT241_ALERT_2_C High   'MainMol' Ueq as Compared to Neighbors of        C16 Check
PLAT242_ALERT_2_C Low    'MainMol' Ueq as Compared to Neighbors of        Cd2 Check
PLAT242_ALERT_2_C Low    'MainMol' Ueq as Compared to Neighbors of         N4 Check
PLAT242_ALERT_2_C Low    'MainMol' Ueq as Compared to Neighbors of         N5 Check
PLAT260_ALERT_2_C Large Average Ueq of Residue Including      O1W      0.177 Check
PLAT260_ALERT_2_C Large Average Ueq of Residue Including      O2W      0.149 Check
PLAT260_ALERT_2_C Large Average Ueq of Residue Including      O3W      0.212 Check
PLAT260_ALERT_2_C Large Average Ueq of Residue Including      O5W      0.138 Check
PLAT260_ALERT_2_C Large Average Ueq of Residue Including      O6W      0.296 Check
PLAT260_ALERT_2_C Large Average Ueq of Residue Including      O7W      0.180 Check
PLAT260_ALERT_2_C Large Average Ueq of Residue Including      O8W      0.116 Check
PLAT260_ALERT_2_C Large Average Ueq of Residue Including      O9W      0.149 Check
PLAT360_ALERT_2_C Short  C(sp3)-C(sp3) Bond  C15      - C16      .       1.40 Ang.
PLAT420_ALERT_2_C D-H Bond Without Acceptor  N3       --H3B      .     Please Check
PLAT420_ALERT_2_C D-H Bond Without Acceptor  N8       --H8A      .     Please Check
PLAT430_ALERT_2_C Short Inter D...A Contact  O1       ..O9W      .       2.86 Ang.
                                                 x,y,z  =      1_555 Check
```

Author Response: There should be a strong hydrogen bond between the two atoms.

```
PLAT430_ALERT_2_C Short Inter D...A Contact  O2W      ..O11      .       2.85 Ang.
                                           1-x,y,1/2-z  =      2_655 Check
```

Author Response: There should be a strong hydrogen bond between the two atoms.

```
PLAT601_ALERT_2_C Unit Cell Contains Solvent Accessible VOIDS of .         64 Ang**3
```

Alert level G

```
FORMU01_ALERT_1_G  There is a discrepancy between the atom counts in the
            _chemical_formula_sum and _chemical_formula_moiety. This is
            usually due to the moiety formula being in the wrong format.
            Atom count from _chemical_formula_sum:   C36 H78 Cd4.5 Ge8 N13 O56 V12
            Atom count from _chemical_formula_moiety:C36 H55 Cd4.5 Ge8 N13 O56 V12
FORMU01_ALERT_2_G  There is a discrepancy between the atom counts in the
            _chemical_formula_sum and the formula from the _atom_site* data.
            Atom count from _chemical_formula_sum:C36 H78 Cd4.5 Ge8 N13 O56 V12
            Atom count from the _atom_site data:  C36 H55 Cd4.5 Ge8 N13 O56 V12
CELLZ01_ALERT_1_G Difference between formula and atom_site contents detected.
CELLZ01_ALERT_1_G WARNING: H atoms missing from atom site list. Is this intentional?
           From the CIF: _cell_formula_units_Z    4
           From the CIF: _chemical_formula_sum  C36 H78 Cd4.50 Ge8 N13 O56 V12
           TEST: Compare cell contents of formula and atom_site data

           atom    Z*formula  cif sites diff
           C        144.00    144.00    0.00
           H        312.00    220.00   92.00
           Cd        18.00     18.00    0.00
           Ge        32.00     32.00    0.00
           N         52.00     52.00    0.00
           O        224.00    224.00    0.00
           V         48.00     48.00    0.00
PLAT002_ALERT_2_G Number of Distance or Angle Restraints on AtSite         11 Note
PLAT003_ALERT_2_G Number of Uiso or Uij Restrained non-H Atoms ...         12 Report
PLAT004_ALERT_5_G Polymeric Structure Found with Maximum Dimension          1 Info
PLAT007_ALERT_5_G Number of Unrefined Donor-H Atoms .............          10 Report
PLAT042_ALERT_1_G Calc. and Reported Moiety Formula Strings Differ     Please Check
PLAT045_ALERT_1_G Calculated and Reported Z Differ by a Factor ...       0.50 Check
PLAT072_ALERT_2_G SHELXL First  Parameter in WGHT  Unusually Large       0.13 Report
PLAT083_ALERT_2_G SHELXL Second Parameter in WGHT  Unusually Large      73.71 Why ?
PLAT172_ALERT_4_G The CIF-Embedded .res File Contains DFIX Records          7 Report
PLAT177_ALERT_4_G The CIF-Embedded .res File Contains DELU Records          1 Report
PLAT186_ALERT_4_G The CIF-Embedded .res File Contains ISOR Records          5 Report
PLAT199_ALERT_1_G Reported _cell_measurement_temperature ..... (K)        293 Check
PLAT200_ALERT_1_G Reported   _diffrn_ambient_temperature ..... (K)        293 Check
PLAT300_ALERT_4_G Atom Site Occupancy of Cd3        Constrained at        0.5 Check
PLAT300_ALERT_4_G Atom Site Occupancy of N6         Constrained at        0.5 Check
PLAT300_ALERT_4_G Atom Site Occupancy of N7         Constrained at        0.5 Check
PLAT300_ALERT_4_G Atom Site Occupancy of N8         Constrained at        0.5 Check
PLAT300_ALERT_4_G Atom Site Occupancy of C17        Constrained at        0.5 Check
PLAT300_ALERT_4_G Atom Site Occupancy of C18        Constrained at        0.5 Check
PLAT300_ALERT_4_G Atom Site Occupancy of C19        Constrained at        0.5 Check
PLAT300_ALERT_4_G Atom Site Occupancy of C20        Constrained at        0.5 Check
PLAT300_ALERT_4_G Atom Site Occupancy of H6A        Constrained at        0.5 Check
PLAT300_ALERT_4_G Atom Site Occupancy of H6B        Constrained at        0.5 Check
PLAT300_ALERT_4_G Atom Site Occupancy of H7         Constrained at        0.5 Check
PLAT300_ALERT_4_G Atom Site Occupancy of H8A        Constrained at        0.5 Check
PLAT300_ALERT_4_G Atom Site Occupancy of H8B        Constrained at        0.5 Check
PLAT300_ALERT_4_G Atom Site Occupancy of H17A       Constrained at        0.5 Check
PLAT300_ALERT_4_G Atom Site Occupancy of H17B       Constrained at        0.5 Check
PLAT300_ALERT_4_G Atom Site Occupancy of H18A       Constrained at        0.5 Check
PLAT300_ALERT_4_G Atom Site Occupancy of H18B       Constrained at        0.5 Check
```

```
PLAT300_ALERT_4_G Atom Site Occupancy of H19A       Constrained at        0.5 Check
PLAT300_ALERT_4_G Atom Site Occupancy of H19B       Constrained at        0.5 Check
PLAT300_ALERT_4_G Atom Site Occupancy of H20A       Constrained at        0.5 Check
PLAT300_ALERT_4_G Atom Site Occupancy of H20B       Constrained at        0.5 Check
PLAT300_ALERT_4_G Atom Site Occupancy of O1W        Constrained at        0.5 Check
PLAT300_ALERT_4_G Atom Site Occupancy of O2W        Constrained at        0.5 Check
PLAT300_ALERT_4_G Atom Site Occupancy of O3W        Constrained at        0.5 Check
PLAT300_ALERT_4_G Atom Site Occupancy of O5W        Constrained at        0.5 Check
PLAT300_ALERT_4_G Atom Site Occupancy of O6W        Constrained at        0.5 Check
PLAT300_ALERT_4_G Atom Site Occupancy of O7W        Constrained at        0.5 Check
PLAT300_ALERT_4_G Atom Site Occupancy of O8W        Constrained at        0.5 Check
PLAT300_ALERT_4_G Atom Site Occupancy of O9W        Constrained at        0.5 Check
PLAT300_ALERT_4_G Atom Site Occupancy of O4W        Constrained at       0.25 Check
PLAT301_ALERT_3_G Main Residue  Disorder .............(Resd  1  )          7% Note
PLAT302_ALERT_4_G Anion/Solvent/Minor-Residue Disorder (Resd  2  )       100% Note
PLAT302_ALERT_4_G Anion/Solvent/Minor-Residue Disorder (Resd  3  )       100% Note
PLAT302_ALERT_4_G Anion/Solvent/Minor-Residue Disorder (Resd  4  )       100% Note
PLAT302_ALERT_4_G Anion/Solvent/Minor-Residue Disorder (Resd  5  )       100% Note
PLAT302_ALERT_4_G Anion/Solvent/Minor-Residue Disorder (Resd  6  )       100% Note
PLAT302_ALERT_4_G Anion/Solvent/Minor-Residue Disorder (Resd  7  )       100% Note
PLAT302_ALERT_4_G Anion/Solvent/Minor-Residue Disorder (Resd  8  )       100% Note
PLAT302_ALERT_4_G Anion/Solvent/Minor-Residue Disorder (Resd  9  )       100% Note
PLAT302_ALERT_4_G Anion/Solvent/Minor-Residue Disorder (Resd 10  )       100% Note
PLAT311_ALERT_2_G Isolated Disordered Oxygen Atom (No H's ?) .....        O1W Check
PLAT311_ALERT_2_G Isolated Disordered Oxygen Atom (No H's ?) .....        O2W Check
PLAT311_ALERT_2_G Isolated Disordered Oxygen Atom (No H's ?) .....        O3W Check
PLAT311_ALERT_2_G Isolated Disordered Oxygen Atom (No H's ?) .....        O5W Check
PLAT311_ALERT_2_G Isolated Disordered Oxygen Atom (No H's ?) .....        O6W Check
PLAT311_ALERT_2_G Isolated Disordered Oxygen Atom (No H's ?) .....        O7W Check
PLAT311_ALERT_2_G Isolated Disordered Oxygen Atom (No H's ?) .....        O8W Check
PLAT311_ALERT_2_G Isolated Disordered Oxygen Atom (No H's ?) .....        O9W Check
PLAT311_ALERT_2_G Isolated Disordered Oxygen Atom (No H's ?) .....        O4W Check
PLAT432_ALERT_2_G Short Inter X...Y Contact  O4W      ..C20              2.74 Ang.
                                         -1/2+x,-1/2+y,z  =      3_445 Check
PLAT764_ALERT_4_G Overcomplete CIF Bond List Detected (Rep/Expd) .       1.15 Ratio
PLAT794_ALERT_5_G Tentative Bond Valency for Cd1       (II)      .       2.03 Info
PLAT794_ALERT_5_G Tentative Bond Valency for Cd2       (II)      .       1.97 Info
PLAT794_ALERT_5_G Tentative Bond Valency for V1        (IV)      .       4.00 Info
PLAT794_ALERT_5_G Tentative Bond Valency for V3        (IV)      .       4.19 Info
PLAT794_ALERT_5_G Tentative Bond Valency for V4        (IV)      .       3.99 Info
PLAT794_ALERT_5_G Tentative Bond Valency for V5        (IV)      .       4.21 Info
PLAT794_ALERT_5_G Tentative Bond Valency for V6        (IV)      .       4.12 Info
PLAT794_ALERT_5_G Tentative Bond Valency for V7        (IV)      .       4.12 Info
PLAT802_ALERT_4_G CIF Input Record(s) with more than 80 Characters          2 Info
PLAT860_ALERT_3_G Number of Least-Squares Restraints ............          74 Note
PLAT941_ALERT_3_G Average HKL Measurement Multiplicity ..........         3.9 Low
```

---

0 **ALERT level A** = Most likely a serious problem - resolve or explain
11 **ALERT level B** = A potentially serious problem, consider carefully
35 **ALERT level C** = Check. Ensure it is not caused by an omission or oversight
79 **ALERT level G** = General information/check it is not something unexpected

11 ALERT type 1 CIF construction/syntax error, inconsistent or missing data

```
 50 ALERT type 2 Indicator that the structure model may be wrong or deficient
  5 ALERT type 3 Indicator that the structure quality may be low
 49 ALERT type 4 Improvement, methodology, query or suggestion
 10 ALERT type 5 Informative message, check
```

Datablock: k3

Bond precision: C-C = 0.0350 A Wavelength=0.71073

Cell:	a=17.9913(3)	b=23.6117(4)	c=23.9327(4)
	alpha=90	beta=91.7290(13)	gamma=90
Temperature:	293 K		

	Calculated	Reported
Volume	10162.1(3)	10162.1(3)
Space group	P 21/n	P 1 21/n 1
Hall group	-P 2yn	-P 2yn
Moiety formula	2(C18 H44 Cd5 Ge6 N18 O48 V15), 2(C6 H24 Cd N6), 2(O0.50), 2(O0	C18 H44 Cd5 Ge6 N18 O48 V15, 6.5(O), C6 H24 Cd N6
Sum formula	C48 H136 Cd12 Ge12 N48 O109 V30	C24 H109 Cd6 Ge6 N24 O54.50 V15
Mr	6878.49	3480.39
Dx,g cm-3	2.248	2.275
Z	2	4
Mu (mm-1)	4.366	4.367
F000	6564.0	6728.0
F000’	6568.85	
h,k,lmax	24,32,32	23,31,32
Nref	27394	23431
Tmin,Tmax	0.384,0.436	0.312,1.000
Tmin’	0.352	

Correction method= # Reported T Limits: Tmin=0.312 Tmax=1.000
AbsCorr = MULTI-SCAN

Data completeness= 0.855 Theta(max)= 29.145

R(reflections)= 0.0780(16299) wR2(reflections)= 0.2417(23431)

S = 1.027 Npar= 1207

The following ALERTS were generated. Each ALERT has the format
test-name_ALERT_alert-type_alert-level.
Click on the hyperlinks for more details of the test.

Alert level B

PLAT043_ALERT_1_B Calculated and Reported Mol. Weight Differ by .. 82.29 Check

Author Response: The hydrogens of all the water molecules was not added.

PLAT097_ALERT_2_B Large Reported Max. (Positive) Residual Density 7.33 eA-3

Author Response: the peak which has no chemical significance, is only about 1.099 Ang away from Cd1. there should be some disorder associated with Cd1, however,the disorder is hardly to be modeled.

PLAT306_ALERT_2_B Isolated Oxygen Atom (H-atoms Missing ?) .... O5W Check

Author Response: The hydrogens of the water molecule was not added.

PLAT306_ALERT_2_B Isolated Oxygen Atom (H-atoms Missing ?) .... O6W Check

Author Response: The hydrogens of the water molecule was not added.

PLAT342_ALERT_3_B Low Bond Precision on C-C Bonds .... 0.035 Ang.

Author Response: Most likely resulting from poor quality diffraction data.

PLAT430_ALERT_2_B Short Inter D...A Contact O1 ..O2AA . 2.67 Ang.
-3/2-x,1/2+y,-5/2-z = 2_352 Check

Author Response: There should be a strong hydrogen bond between the two atoms.

PLAT430_ALERT_2_B Short Inter D...A Contact O0AA ..O31 . 2.75 Ang.
1/2+x,1/2-y,1/2+z = 4_666 Check

Author Response: There should be a strong hydrogen bond between the two atoms.

PLAT430_ALERT_2_B Short Inter D...A Contact O0AA ..O4W . 2.77 Ang.
1/2+x,1/2-y,1/2+z = 4_666 Check

Author Response: There should be a strong hydrogen bond between the two atoms.

```
PLAT430_ALERT_2_B Short Inter D...A Contact  O1W      ..O25      .       2.82 Ang.
                                                x,y,z  =      1_555 Check
```

Author Response: There should be a strong hydrogen bond between the two atoms.

```
PLAT430_ALERT_2_B Short Inter D...A Contact  O1W      ..O25      .       2.82 Ang.
                                        -2-x,1-y,-2-z  =      3_363 Check
```

Author Response: There should be a strong hydrogen bond between the two atoms.

```
PLAT430_ALERT_2_B Short Inter D...A Contact  O1AA     ..O17      .       2.67 Ang.
                                                x,y,z  =      1_555 Check
```

Author Response: There should be a strong hydrogen bond between the two atoms.

```
PLAT430_ALERT_2_B Short Inter D...A Contact  O2W      ..O5       .       2.59 Ang.
                                                x,y,z  =      1_555 Check
```

Author Response: There should be a strong hydrogen bond between the two atoms.

```
PLAT430_ALERT_2_B Short Inter D...A Contact  O3W      ..O34      .       2.77 Ang.
                                                x,y,z  =      1_555 Check
```

Author Response: There should be a strong hydrogen bond between the two atoms.

```
PLAT430_ALERT_2_B Short Inter D...A Contact  O3AA     ..O48      .       2.84 Ang.
                                 -3/2-x,-1/2+y,-5/2-z  =      2_342 Check
```

Author Response: There should be a strong hydrogen bond between the two atoms.

```
PLAT430_ALERT_2_B Short Inter D...A Contact  O4W      ..O4W      .       2.62 Ang.
                                        -2-x,1-y,-3-z  =      3_362 Check
```

Author Response: There should be a strong hydrogen bond between the two atoms.

```
PLAT430_ALERT_2_B Short Inter D...A Contact  O4W      ..O9       .       2.76 Ang.
                                                x,y,z  =      1_555 Check
```

Author Response: There should be a strong hydrogen bond between the two atoms.

```
PLAT430_ALERT_2_B Short Inter D...A Contact  O5W      ..O5W      .       2.67 Ang.
                                         -2-x,-y,-3-z  =      3_352 Check
```

Author Response: There should be a strong hydrogen bond between the two atoms.

Alert level C

```
DIFMN02_ALERT_2_C  The minimum difference density is < -0.1*ZMAX*0.75
            _refine_diff_density_min given =     -3.675
            Test value =     -3.600
DIFMN03_ALERT_1_C  The minimum difference density is < -0.1*ZMAX*0.75
            The relevant atom site should be identified.
DIFMX02_ALERT_1_C  The maximum difference density is > 0.1*ZMAX*0.75
            The relevant atom site should be identified.
PLAT041_ALERT_1_C Calc. and Reported SumFormula    Strings  Differ     Please Check
PLAT068_ALERT_1_C Reported F000 Differs from Calcd (or Missing)...     Please Check
PLAT098_ALERT_2_C Large Reported Min.  (Negative) Residual Density      -3.67 eA-3
PLAT241_ALERT_2_C High   'MainMol' Ueq as Compared to Neighbors of        N13 Check
PLAT241_ALERT_2_C High   'MainMol' Ueq as Compared to Neighbors of        N15 Check
PLAT241_ALERT_2_C High   'MainMol' Ueq as Compared to Neighbors of        N16 Check
PLAT241_ALERT_2_C High   'MainMol' Ueq as Compared to Neighbors of        N21 Check
PLAT241_ALERT_2_C High   'MainMol' Ueq as Compared to Neighbors of         C8 Check
PLAT241_ALERT_2_C High   'MainMol' Ueq as Compared to Neighbors of        C11 Check
PLAT241_ALERT_2_C High   'MainMol' Ueq as Compared to Neighbors of        C12 Check
PLAT241_ALERT_2_C High   'MainMol' Ueq as Compared to Neighbors of        C17 Check
PLAT241_ALERT_2_C High   'MainMol' Ueq as Compared to Neighbors of        C20 Check
PLAT241_ALERT_2_C High   'MainMol' Ueq as Compared to Neighbors of         C1 Check
PLAT241_ALERT_2_C High   'MainMol' Ueq as Compared to Neighbors of         C2 Check
PLAT241_ALERT_2_C High   'MainMol' Ueq as Compared to Neighbors of         C3 Check
PLAT241_ALERT_2_C High   'MainMol' Ueq as Compared to Neighbors of         C4 Check
PLAT241_ALERT_2_C High   'MainMol' Ueq as Compared to Neighbors of         C5 Check
PLAT242_ALERT_2_C Low    'MainMol' Ueq as Compared to Neighbors of        Cd1 Check
PLAT242_ALERT_2_C Low    'MainMol' Ueq as Compared to Neighbors of        Cd3 Check
PLAT242_ALERT_2_C Low    'MainMol' Ueq as Compared to Neighbors of        Cd4 Check
PLAT242_ALERT_2_C Low    'MainMol' Ueq as Compared to Neighbors of        Cd5 Check
PLAT242_ALERT_2_C Low    'MainMol' Ueq as Compared to Neighbors of        Cd6 Check
PLAT242_ALERT_2_C Low    'MainMol' Ueq as Compared to Neighbors of        O24 Check
PLAT242_ALERT_2_C Low    'MainMol' Ueq as Compared to Neighbors of         N2 Check
PLAT242_ALERT_2_C Low    'MainMol' Ueq as Compared to Neighbors of         N3 Check
PLAT260_ALERT_2_C Large Average Ueq of Residue Including      O0AA      0.122 Check
PLAT260_ALERT_2_C Large Average Ueq of Residue Including       O1W      0.181 Check
PLAT260_ALERT_2_C Large Average Ueq of Residue Including      O1AA      0.170 Check
PLAT260_ALERT_2_C Large Average Ueq of Residue Including       O2W      0.128 Check
PLAT260_ALERT_2_C Large Average Ueq of Residue Including      O2AA      0.210 Check
PLAT260_ALERT_2_C Large Average Ueq of Residue Including       O3W      0.154 Check
PLAT260_ALERT_2_C Large Average Ueq of Residue Including      O3AA      0.142 Check
PLAT260_ALERT_2_C Large Average Ueq of Residue Including       O4W      0.133 Check
PLAT260_ALERT_2_C Large Average Ueq of Residue Including       O5W      0.159 Check
PLAT260_ALERT_2_C Large Average Ueq of Residue Including       O6W      0.189 Check
PLAT260_ALERT_2_C Large Average Ueq of Residue Including       O7W      0.131 Check
PLAT360_ALERT_2_C Short  C(sp3)-C(sp3) Bond  C7       - C8       .       1.42 Ang.
PLAT360_ALERT_2_C Short  C(sp3)-C(sp3) Bond  C21      - C22      .       1.42 Ang.
PLAT360_ALERT_2_C Short  C(sp3)-C(sp3) Bond  C1       - C2       .       1.36 Ang.
PLAT360_ALERT_2_C Short  C(sp3)-C(sp3) Bond  C3       - C4       .       1.40 Ang.
PLAT360_ALERT_2_C Short  C(sp3)-C(sp3) Bond  C5       - C6       .       1.36 Ang.
PLAT410_ALERT_2_C Short Intra H...H Contact  H1C      ..H2D      .       1.93 Ang.
                                                      x,y,z  =      1_555 Check
PLAT410_ALERT_2_C Short Intra H...H Contact  H1D      ..H2C      .       1.94 Ang.
                                                      x,y,z  =      1_555 Check
PLAT414_ALERT_2_C Short Intra D-H..H-X       H7A      ..H7D              1.97 Ang.
                                                      x,y,z  =      1_555 Check
```

```
PLAT414_ALERT_2_C Short Intra D-H..H-X       H7B      ..H7C             1.97 Ang.
                                                      x,y,z  =      1_555 Check
PLAT414_ALERT_2_C Short Intra D-H..H-X       H2A      ..H2C             1.95 Ang.
                                                      x,y,z  =      1_555 Check
PLAT414_ALERT_2_C Short Intra D-H..H-X       H2B      ..H2D             1.94 Ang.
                                                      x,y,z  =      1_555 Check
PLAT420_ALERT_2_C D-H Bond Without Acceptor  N1       --H1A      .    Please Check
PLAT420_ALERT_2_C D-H Bond Without Acceptor  N2       --H2A      .    Please Check
PLAT420_ALERT_2_C D-H Bond Without Acceptor  N3       --H3A      .    Please Check
PLAT420_ALERT_2_C D-H Bond Without Acceptor  N7       --H7A      .    Please Check
PLAT420_ALERT_2_C D-H Bond Without Acceptor  N8       --H8B      .    Please Check
PLAT420_ALERT_2_C D-H Bond Without Acceptor  N11      --H11A     .    Please Check
PLAT420_ALERT_2_C D-H Bond Without Acceptor  N12      --H12A     .    Please Check
PLAT420_ALERT_2_C D-H Bond Without Acceptor  N21      --H21B     .    Please Check
PLAT420_ALERT_2_C D-H Bond Without Acceptor  N23      --H23A     .    Please Check
PLAT420_ALERT_2_C D-H Bond Without Acceptor  N23      --H23B     .    Please Check
PLAT420_ALERT_2_C D-H Bond Without Acceptor  N25      --H25B     .    Please Check
PLAT430_ALERT_2_C Short Inter D...A Contact  O3W      ..O17      .      2.85 Ang.
                                                      x,y,z  =      1_555 Check
```

Author Response: There should be a strong hydrogen bond between the two atoms.

Alert level G

```
FORMU01_ALERT_1_G  There is a discrepancy between the atom counts in the
            _chemical_formula_sum and _chemical_formula_moiety. This is
            usually due to the moiety formula being in the wrong format.
            Atom count from _chemical_formula_sum:   C24 H109 Cd6 Ge6 N24 O54.5 V1
            Atom count from _chemical_formula_moiety:C24 H68 Cd6 Ge6 N24 O54.5 V15
FORMU01_ALERT_2_G  There is a discrepancy between the atom counts in the
            _chemical_formula_sum and the formula from the _atom_site* data.
            Atom count from _chemical_formula_sum:C24 H109 Cd6 Ge6 N24 O54.5 V15
            Atom count from the _atom_site data:  C24 H68 Cd6 Ge6 N24 O54.5 V15
CELLZ01_ALERT_1_G Difference between formula and atom_site contents detected.
CELLZ01_ALERT_1_G WARNING: H atoms missing from atom site list. Is this intentional?
           From the CIF: _cell_formula_units_Z    4
           From the CIF: _chemical_formula_sum  C24 H109 Cd6 Ge6 N24 O54.50 V15
           TEST: Compare cell contents of formula and atom_site data

           atom    Z*formula  cif sites diff
           C         96.00     96.00    0.00
           H        436.00    272.00  164.00
           Cd        24.00     24.00    0.00
           Ge        24.00     24.00    0.00
           N         96.00     96.00    0.00
           O        218.00    218.00    0.00
           V         60.00     60.00    0.00
PLAT002_ALERT_2_G Number of Distance or Angle Restraints on AtSite         19 Note
PLAT003_ALERT_2_G Number of Uiso or Uij Restrained non-H Atoms ...         33 Report
PLAT004_ALERT_5_G Polymeric Structure Found with Maximum Dimension          3 Info
PLAT007_ALERT_5_G Number of Unrefined Donor-H Atoms .............          34 Report
PLAT042_ALERT_1_G Calc. and Reported Moiety Formula Strings Differ     Please Check
PLAT045_ALERT_1_G Calculated and Reported Z Differ by a Factor ...       0.50 Check
PLAT072_ALERT_2_G SHELXL First  Parameter in WGHT  Unusually Large       0.12 Report
```

```
PLAT083_ALERT_2_G SHELXL Second Parameter in WGHT  Unusually Large      97.08 Why ?
PLAT172_ALERT_4_G The CIF-Embedded .res File Contains DFIX Records          5 Report
PLAT178_ALERT_4_G The CIF-Embedded .res File Contains SIMU Records          8 Report
PLAT186_ALERT_4_G The CIF-Embedded .res File Contains ISOR Records          4 Report
PLAT199_ALERT_1_G Reported _cell_measurement_temperature ..... (K)        293 Check
PLAT200_ALERT_1_G Reported   _diffrn_ambient_temperature ..... (K)        293 Check
PLAT300_ALERT_4_G Atom Site Occupancy of O0AA       Constrained at        0.5 Check
PLAT300_ALERT_4_G Atom Site Occupancy of O1W        Constrained at        0.5 Check
PLAT300_ALERT_4_G Atom Site Occupancy of O1AA       Constrained at        0.5 Check
PLAT300_ALERT_4_G Atom Site Occupancy of O2W        Constrained at        0.5 Check
PLAT300_ALERT_4_G Atom Site Occupancy of O2AA       Constrained at        0.5 Check
PLAT300_ALERT_4_G Atom Site Occupancy of O3W        Constrained at        0.5 Check
PLAT300_ALERT_4_G Atom Site Occupancy of O3AA       Constrained at        0.5 Check
PLAT300_ALERT_4_G Atom Site Occupancy of O4W        Constrained at        0.5 Check
PLAT300_ALERT_4_G Atom Site Occupancy of O7W        Constrained at        0.5 Check
PLAT302_ALERT_4_G Anion/Solvent/Minor-Residue Disorder (Resd  3  )       100% Note
PLAT302_ALERT_4_G Anion/Solvent/Minor-Residue Disorder (Resd  4  )       100% Note
PLAT302_ALERT_4_G Anion/Solvent/Minor-Residue Disorder (Resd  5  )       100% Note
PLAT302_ALERT_4_G Anion/Solvent/Minor-Residue Disorder (Resd  6  )       100% Note
PLAT302_ALERT_4_G Anion/Solvent/Minor-Residue Disorder (Resd  7  )       100% Note
PLAT302_ALERT_4_G Anion/Solvent/Minor-Residue Disorder (Resd  8  )       100% Note
PLAT302_ALERT_4_G Anion/Solvent/Minor-Residue Disorder (Resd  9  )       100% Note
PLAT302_ALERT_4_G Anion/Solvent/Minor-Residue Disorder (Resd 10  )       100% Note
PLAT302_ALERT_4_G Anion/Solvent/Minor-Residue Disorder (Resd 13  )       100% Note
PLAT311_ALERT_2_G Isolated Disordered Oxygen Atom (No H's ?) .....       O0AA Check
PLAT311_ALERT_2_G Isolated Disordered Oxygen Atom (No H's ?) .....        O1W Check
PLAT311_ALERT_2_G Isolated Disordered Oxygen Atom (No H's ?) .....       O1AA Check
PLAT311_ALERT_2_G Isolated Disordered Oxygen Atom (No H's ?) .....        O2W Check
PLAT311_ALERT_2_G Isolated Disordered Oxygen Atom (No H's ?) .....       O2AA Check
PLAT311_ALERT_2_G Isolated Disordered Oxygen Atom (No H's ?) .....        O3W Check
PLAT311_ALERT_2_G Isolated Disordered Oxygen Atom (No H's ?) .....       O3AA Check
PLAT311_ALERT_2_G Isolated Disordered Oxygen Atom (No H's ?) .....        O4W Check
PLAT311_ALERT_2_G Isolated Disordered Oxygen Atom (No H's ?) .....        O7W Check
PLAT343_ALERT_2_G Unusual sp?      Angle Range in Main Residue for        C13 Check
PLAT343_ALERT_2_G Unusual sp?      Angle Range in Main Residue for        C14 Check
PLAT343_ALERT_2_G Unusual sp?      Angle Range in Main Residue for        C15 Check
PLAT343_ALERT_2_G Unusual sp?      Angle Range in Main Residue for        C18 Check
PLAT343_ALERT_2_G Unusual sp?      Angle Range in Main Residue for        C19 Check
PLAT343_ALERT_2_G Unusual sp?      Angle Range in Main Residue for        C20 Check
PLAT343_ALERT_2_G Unusual sp?      Angle Range in Main Residue for        C25 Check
PLAT343_ALERT_2_G Unusual sp3      Angle Range in Main Residue for         C2 Check
PLAT720_ALERT_4_G Number of Unusual/Non-Standard Labels ..........          4 Note
PLAT764_ALERT_4_G Overcomplete CIF Bond List Detected (Rep/Expd) .       1.14 Ratio
PLAT794_ALERT_5_G Tentative Bond Valency for Cd1       (II)      .       2.00 Info
PLAT794_ALERT_5_G Tentative Bond Valency for Cd2       (II)      .       1.97 Info
PLAT794_ALERT_5_G Tentative Bond Valency for Cd3       (II)      .       2.47 Info
PLAT794_ALERT_5_G Tentative Bond Valency for Cd4       (II)      .       1.95 Info
PLAT794_ALERT_5_G Tentative Bond Valency for Cd5       (II)      .       2.08 Info
PLAT794_ALERT_5_G Tentative Bond Valency for Cd6       (II)      .       2.04 Info
PLAT794_ALERT_5_G Tentative Bond Valency for V1        (IV)      .       4.15 Info
PLAT794_ALERT_5_G Tentative Bond Valency for V2        (IV)      .       4.09 Info
PLAT794_ALERT_5_G Tentative Bond Valency for V3        (IV)      .       4.14 Info
PLAT794_ALERT_5_G Tentative Bond Valency for V4        (IV)      .       4.16 Info
PLAT794_ALERT_5_G Tentative Bond Valency for V5        (IV)      .       4.18 Info
PLAT794_ALERT_5_G Tentative Bond Valency for V6        (IV)      .       4.11 Info
PLAT794_ALERT_5_G Tentative Bond Valency for V7        (IV)      .       4.08 Info
PLAT794_ALERT_5_G Tentative Bond Valency for V8        (IV)      .       4.10 Info
```

```
PLAT794_ALERT_5_G Tentative Bond Valency for V9        (IV)      .       4.12 Info
PLAT794_ALERT_5_G Tentative Bond Valency for V10       (IV)      .       4.05 Info
PLAT794_ALERT_5_G Tentative Bond Valency for V11       (IV)      .       4.14 Info
PLAT794_ALERT_5_G Tentative Bond Valency for V12       (IV)      .       4.14 Info
PLAT794_ALERT_5_G Tentative Bond Valency for V13       (IV)      .       4.13 Info
PLAT794_ALERT_5_G Tentative Bond Valency for V14       (IV)      .       4.14 Info
PLAT794_ALERT_5_G Tentative Bond Valency for V15       (IV)      .       4.06 Info
PLAT802_ALERT_4_G CIF Input Record(s) with more than 80 Characters          1 Info
PLAT860_ALERT_3_G Number of Least-Squares Restraints .............        259 Note
PLAT941_ALERT_3_G Average HKL Measurement Multiplicity ..........         2.3 Low
```

```
  0 ALERT level A = Most likely a serious problem - resolve or explain
 17 ALERT level B = A potentially serious problem, consider carefully
 62 ALERT level C = Check. Ensure it is not caused by an omission or oversight
 78 ALERT level G = General information/check it is not something unexpected

 12 ALERT type 1 CIF construction/syntax error, inconsistent or missing data
 95 ALERT type 2 Indicator that the structure model may be wrong or deficient
  3 ALERT type 3 Indicator that the structure quality may be low
 24 ALERT type 4 Improvement, methodology, query or suggestion
 23 ALERT type 5 Informative message, check
```

It is advisable to attempt to resolve as many as possible of the alerts in all categories. Often the minor alerts point to easily fixed oversights, errors and omissions in your CIF or refinement strategy, so attention to these fine details can be worthwhile. In order to resolve some of the more serious problems it may be necessary to carry out additional measurements or structure refinements. However, the purpose of your study may justify the reported deviations and the more serious of these should normally be commented upon in the discussion or experimental section of a paper or in the "special_details" fields of the CIF. checkCIF was carefully designed to identify outliers and unusual parameters, but every test has its limitations and alerts that are not important in a particular case may appear. Conversely, the absence of alerts does not guarantee there are no aspects of the results needing attention. It is up to the individual to critically assess their own results and, if necessary, seek expert advice.

Publication of your CIF in IUCr journals

A basic structural check has been run on your CIF. These basic checks will be run on all CIFs submitted for publication in IUCr journals (*Acta Crystallographica*, *Journal of Applied Crystallography*, *Journal of Synchrotron Radiation*); however, if you intend to submit to *Acta Crystallographica Section C* or *E* or *IUCrData*, you should make sure that full publication checks are run on the final version of your CIF prior to submission.

Publication of your CIF in other journals

Please refer to the *Notes for Authors* of the relevant journal for any special instructions relating to CIF submission.

PLATON version of 13/07/2021; check.def file version of 13/07/2021

Datablock k1 - ellipsoid plot

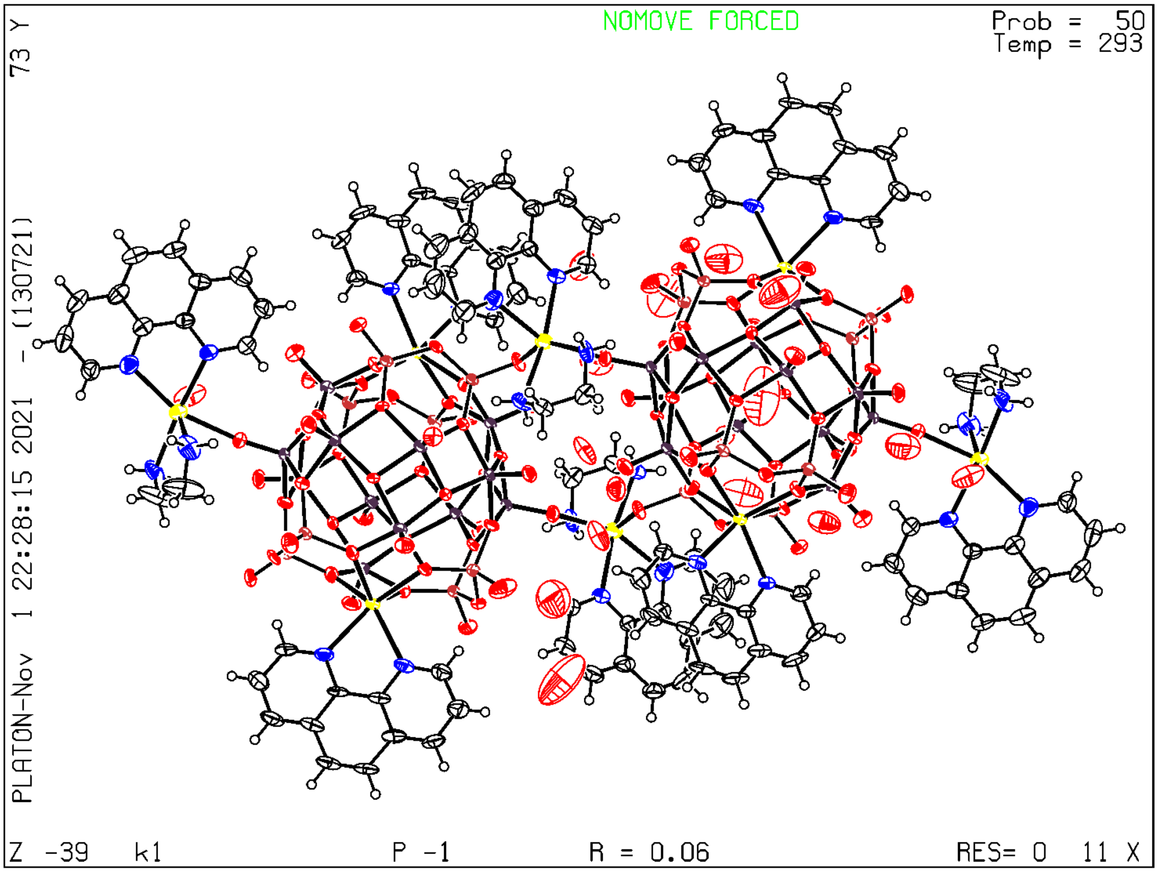

Datablock k2 - ellipsoid plot

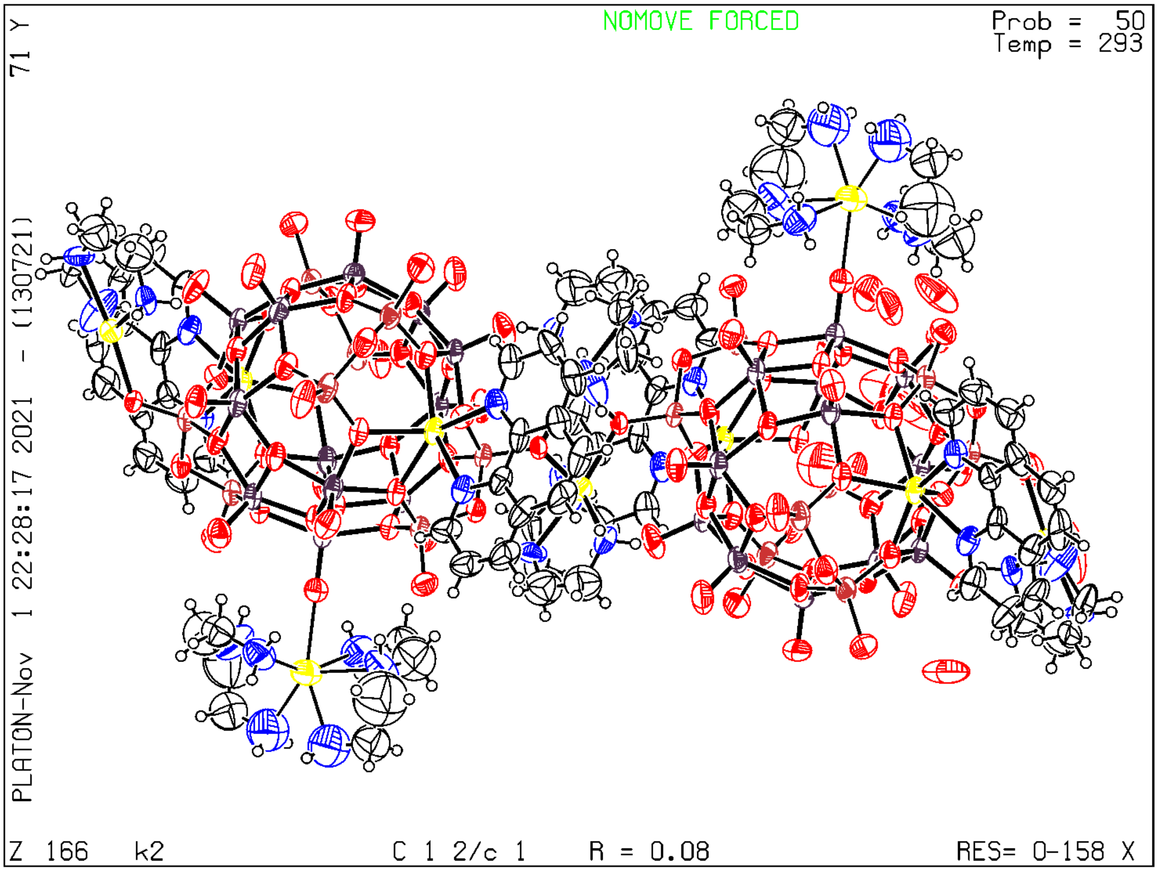

Datablock k3 - ellipsoid plot

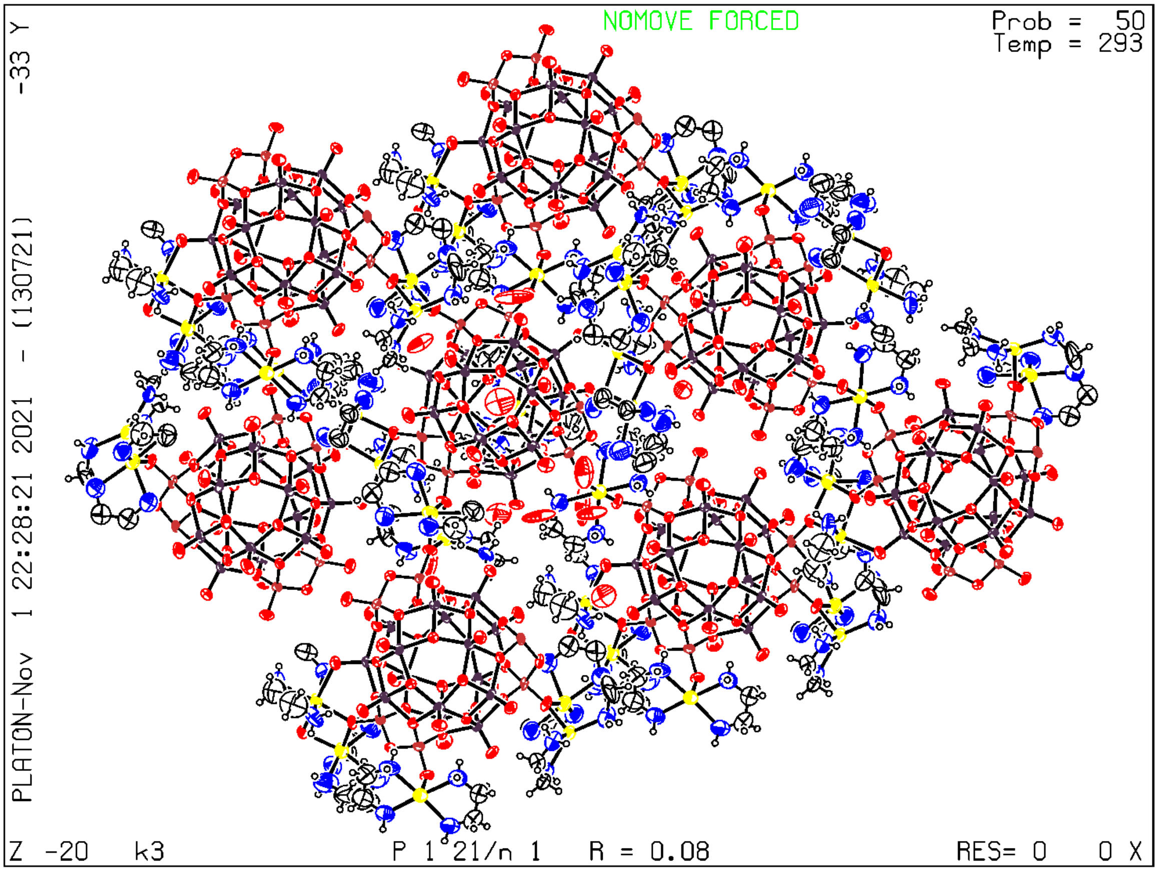

Supplement: Supplementary file 1 [file molecules-27-04424-s001.zip › checkcif.pdf]
