# Supplementary material for: First Organic–Inorganic Hybrid Compounds Formed by Ge-V-O Clusters and Transition Metal Complexes of Aromatic Organic Ligands
Source: Molecules. 2022 Jul 11;27(14):4424. doi: 10.3390/molecules27144424 (PMC9323094; doi:10.3390/molecules27144424)
Supplement: Supplementary file 1 [file molecules-27-04424-s001.zip › supporting information.pdf]

Supporting Information

First Organic–Inorganic Hybrid Compounds Formed by Ge-V-O Clusters and Transition Metal Complexes of Aromatic Organic Ligands

Hai-Yang Guo [1,2], Hui Qi [3], Xiao Zhang [4] and Xiao-Bing Cui [1,*]

1 State Key Laboratory of Inorganic Synthesis and Preparative Chemistry and College of Chemistry, Jilin University, Changchun 130021, China; guohy@zjxu.edu.cn
2 College of Biological, Chemical Science and Engineering, Jiaxing University, Jiaxing 314001, China
3 The Second Hospital of Jilin University, Changchun 130021, China; qihui1977@sohu.com
4 MIIT Key Laboratory of Critical Materials Technology for New Energy Conversion and Storage, School of Chemistry and Chemical Engineering, Harbin Institute of Technology, 150001 Harbin, China; zhangx@hit.edu.cn
* Correspondence: cuixb@mail.jlu.edu.cn

Contents

A. Bond valence sum calculations S2

B. IR spectra S5

C. Powder X-ray diffraction patterns S7

D. UV-vis spectra S9

E. ESR spectra S11

F. The comparisons of the catalytic oxidation of styrene S13

G. X-ray diffraction patterns FT-IR spectra of compound 3 after the three cycles S14

H. References S14

A. Bond valence sum calculations

Table S1. Bond valence sum calculations for Ge, V and O in compounds **1**-**3**.

Compound **1**		Compound **2**		Compound **3**	
Ge(8)	3.97	Ge(1)	4.04	Ge(6)	4.04
Ge(7)	4.01	Ge(2)	4.00	Ge(8)	4.07
Ge(6)	3.98	Ge(3)	3.91	Ge(9)	4.09
Ge(5)	4.00	Ge(4)	4.06	Ge(10)	4.02
Ge(4)	4.03			Ge(11)	4.04
Ge(3)	4.02			Ge(12)	4.03
Ge(2)	4.03				
Ge(1)	4.03				
Equation S=exp[-(r-1.748)/0.37] (**1**) for BVS of Ge.[1]					
V(12)	4.18	V(1)	4.00	V(15)	4.07
V(11)	4.18	V(2)	4.17	V(14)	4.13
V(10)	4.17	V(3)	4.15	V(13)	4.13
V(9)	4.00	V(4)	3.99	V(12)	4.14
V(8)	4.05	V(5)	4.21	V(11)	4.14
V(7)	4.14	V(6)	4.12	V(10)	4.05
V(6)	4.14	V(7)	4.12	V(9)	4.12
V(5)	4.08			V(8)	4.10
V(4)	4.13			V(7)	4.07
V(3)	4.06			V(6)	4.11
V(2)	4.02			V(5)	4.18
V(1)	4.06			V(4)	4.17
				V(3)	4.13
				V(2)	4.10
				V(1)	4.15
Equation S=exp[-(r-1.784)/0.37] (**1**) for BVS of V.[1]					
O(1) Ge_t	1.02	O(1) V_t	1.60	O(1) GeCd	1.57
O(2) 2V1Ge	2.09	O(2) V_t	1.55	O(2) 2CdGe	1.94
O(3)V_t	1.67	O(3) 3V	2.03	O(3) 2CdGe	2.01
O(4)V_t	1.60	O(4) 3V	1.98	O(4)V_t	1.60
O(5) V_t	1.67	O(5) V_t	1.66	O(5) GeCd	1.57
O(6) Ge_t	1.01	O(6) V_t	1.65	O(6) 2V1Ge	2.15
O(7) V_t	1.62	O(7) 2V1Ge	2.14	O(7) V_t	1.57
O(8) V_t	1.56	O(8) 3V	1.99	O(8) V_t	1.60
O(9) V_t	1.61	O(9) V_t	1.55	O(9) V_t	1.65
O(10) V_t	1.59	O(10) Ge_t2Cd	1.85	O(10) V_t	1.60
O(11) V_t	1.63	O(11) V_t	1.59	O(11) 3V	1.96
O(12) V_t	1.59	O(12) VGeCd	2.01	O(12) 2Ge	1.93
O(13) Ge_tCd	1.38	O(13) 3V	1.97	O(13) 3V	2.09
O(14) Ge_t	1.03	O(14) 2V1Ge	2.13	O(14) 3V	2.06
O(15) Ge_t	1.01	O(16) VGeCd	2.00	O(15) 2V1Ge	2.13

O(16) 2V1Ge	2.08	O(17) V_t	1.72	O(16) 2CdGe	1.90
O(17) CdVGe	1.98	O(18) 2V1Ge	2.12	O(17) V_t	1.62
O(18) CdVGe	1.95	O(19) VGeCd	2.03	O(18) 2V1Ge	2.11
O(19) V_t	1.62	O(20) 2V1Ge	2.13	O(19) 2V1Ge	2.17
O(20) 3V	2.00	O(21) 2Ge	1.84	O(20) 2Ge	1.87
O(21) Ge_t	1.02	O(22) Ge_t	1.06	O(21) VCd	1.66
O(22) V_t	1.58	O(23) Ge_t	0.94	O(22) 2V1Ge	2.18
O(23) 2Ge	1.92	O(24) VGeCd	1.97	O(23) 3V	1.97
O(24) Ge_t	1.01	O(25) 2Ge	1.85	O(24) 2CdGe	1.81
O(25) 2V1Ge	2.14	O(26) Ge_t	1.01	O(25) V_t	1.54
O(26) 2Ge	1.92			O(26) 2Ge	1.87
O(27) CdVGe	1.99			O(27) 3V	1.96
O(28) 2V1Ge	2.16			O(28) 3V	2.11
O(29) CdVGe	2.01			O(29) 3V	1.98
O(30) 2Ge	1.92			O(30) 3V	2.01
O(31) 3V	2.02			O(31) V_t	1.56
O(32) Ge_t	1.02			O(32) 2V1Ge	2.00
O(33) CdVGe	1.96			O(33) V_t	1.60
O(34) 2V1Ge	2.15			O(34) V_t	1.60
O(35) V_tCd	1.77			O(35) 2V1Ge	2.06
O(36) 2Ge	1.93			O(36) 3V	1.94
O(37) 3V	2.08			O(37) V_t	1.62
O(38) CdVGe	1.98			O(38) 2V1Ge	2.13
O(39) 2V1Ge	2.15			O(39) 2V1Ge	2.16
O(40) 2V1Ge	2.07			O(40) 2V1Ge	2.10
O(41) 3V	2.01			O(41) 3V	2.06
O(42) 3V	1.98			O(42) 2V1Ge	2.04
O(43) 2V1Ge	2.14			O(43) 2V1Ge	2.05
O(44) 3V	1.97			O(44) 3V	2.07
O(45) CdVGe	2.01			O(45) V_t	1.64
O(46) 3V	1.97			O(46) V_t	1.58
O(47) CdVGe	1.97			O(47) 3V	2.06
O(48) 3V	2.01			O(48) V_t	1.63

Equation S=exp[-(r-1.784)/0.37] (**1**) for BVS of V.[1]

Equation S=exp[-(r-1.748)/0.37] (**1**) for BVS of Ge.[1]

Equation S=exp[-(r-1.904)/0.37] (**1**) for BVS of Ge.[1]

All the three compounds, $[Cd(phen)(en)]_2[Cd_2(phen)_2V_{12}O_{40}Ge_8(OH)_8(H_2O)]\cdot 12.5H_2O$ (**1**), $[Cd(DETA)]_2[Cd(DETA)_2]_{0.5}[Cd_2(phen)_2V_{12}O_{41}Ge_8(OH)_7(0.5H_2O)]\cdot 7.5H_2O$ (**2**) and $[Cd(en)_3]\{[Cd(\eta_2\text{-}en)_2]_3[Cd(\eta_2\text{-}en)(\eta_1\text{-}en)(\eta_2\text{-}en)Cd][Ge_6V_{15}O_{48}(H_2O)]\}\cdot 5.5H_2O$ (**3**) were synthesized under very strong basic conditions. For coordination complexes and MOFs, it is hard to obtain compounds with hydrogen atoms attached under such severe conditions, but it is not so for polyoxometalates especially for Ge-V clusters and polyoxoniobates (I must say that all the Ge-V clusters and polyoxoniobates are synthesized under strong basic conditions), there are a great number of such examples, part is listed as below: (1). Ozeki, T.; Yamase, T.;

Naruke, H.; Sasaki, Y. X-Ray Structural Characterization of the Protonation Sites in the Dihydrogenhexaniobate Anion. *Bull. Chem. Soc. Jan.*, **1994**, *67,* 3249-3253. (2). Huang, P.; Qin, C.; Su, Z.M.; Xing, Y.; Wang, X.L.; Shao, K.Z.; Lan, Y.Q.; Wang, E.B. Self-Assembly and Photocatalytic Properties of Polyoxoniobates: {$Nb_{24}O_{72}$}, {$Nb_{32}O_{96}$}, and {$K_{12}Nb_{96}O_{288}$} Clusters. *J. Am. Chem. Soc.*, **2012**, *134*, 14004-14010. (3) Nyman, M.; Bonhomme, F.; Alam, T.M.; Bodriguez, M.A.; Cherry, B.R.; Krumhansl, J.L.; Nenoff, T.M.; Sattler, A.M. A General Synthetic Procedure for Heteropolyniobates. *Science.*, **2002**, *297*, 996-998. (4) Zhou, J.; Zhang, J.; Fang, W.H.; Yang, G.Y. A Series of Vanadogermanates from 1D Chain to 3D Framework Built by Ge – V – O Clusters and Transition-Metal-Complex Bridges. *Chem. Eur. J.*, **2010**, *16*, 13253-13261. (5) Zhou, J.; Zhao, J.W.; Wei, Q.; Zhang, J.; Yang, G.Y. Two Tetra-Cd^{II}-Substituted Vanadogermanate Frameworks. *J. Am. Chem. Soc.*, **2014**, *136*, 5065-5071. (6) Wang, J.; Näther, C.; Speldrich, M.; Kögerler P.; Bensch, W. Chain and layer networks of germanato-polyoxovanadates. *CrystEngComm.*, **2013**, *15*, 10238-10245. (7) Wang, J.; Näther, C.; Kögerler, P.; Bensch, W. $[V_{15}Ge_6O_{42}S_6(H_2O)]^{12-}$, a Thiogermanatopolyoxovanadate Cluster Featuringthe Spin Topology of the Molecular Magnet $[V_{15}As_6O_{42}(H_2O)]^{6-}$. *Eur. J. Inorg. Chem.*, **2012**, 1237-1242. (8) Pitzschke, D.; Wang, J.; Hoffmann, R.D.; Pöttgen, R. Bensch, W. Two Compounds Containing the Mixed Germanium -Vanadium Polyoxothioanion $[V_{14}Ge_8O_{42}S_8]^{12-}$. *Angew. Chem. Int. Ed.*, **2006**, *45*, 1305-1308 (references (6)-(8) also contain protons but the protons are not attached to the cluster but to the complexes or organic ligands.) (9) Gao, Y.Z.; Xu, Y.Q.; Huang, K.L.; Han, Z.G.; Hu, C.W. Two three-dimensional {$V_{16}Ge_4$}-based open frameworks stabilized by diverse types of Co^{II}-amine bridges and magnetic properties. *Dalton Trans.*, **2012**, *41*, 6122-6129. (10) Tripathi, A.; Hughbanks, T.; Clearfield, A. The First Framework Solid Composed of Vanadosilicate Clusters. *J. Am. Chem. Soc.,* **2003**, *125*, 10528-10529. (11) Whitfield, T.; Wang, X.; Jacobson, A.J. Vanadogermanate Cluster Anions. *Inorg. Chem.*, **2003**, *42*, 3728-3733.

B. IR spectra

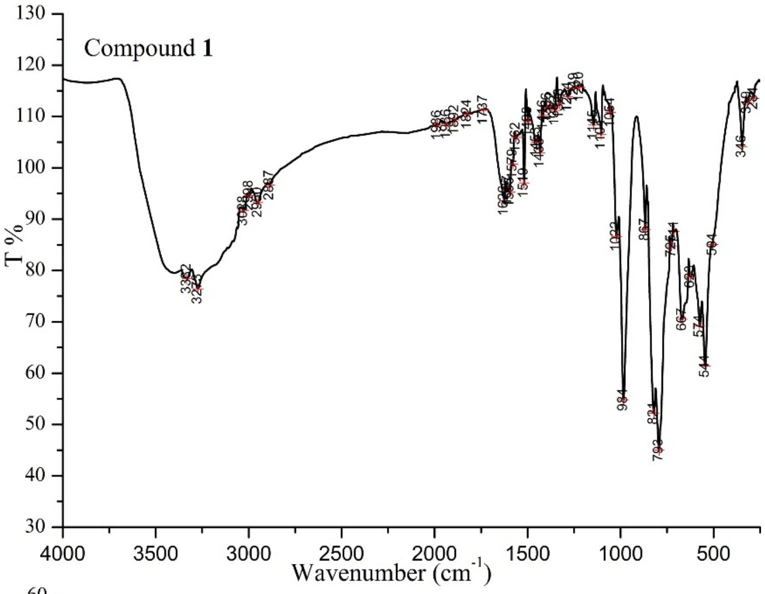
Compound 1
T %
Wavenumber (cm-1)
1737
1022
984
867
824
793
667
574
544
504
346

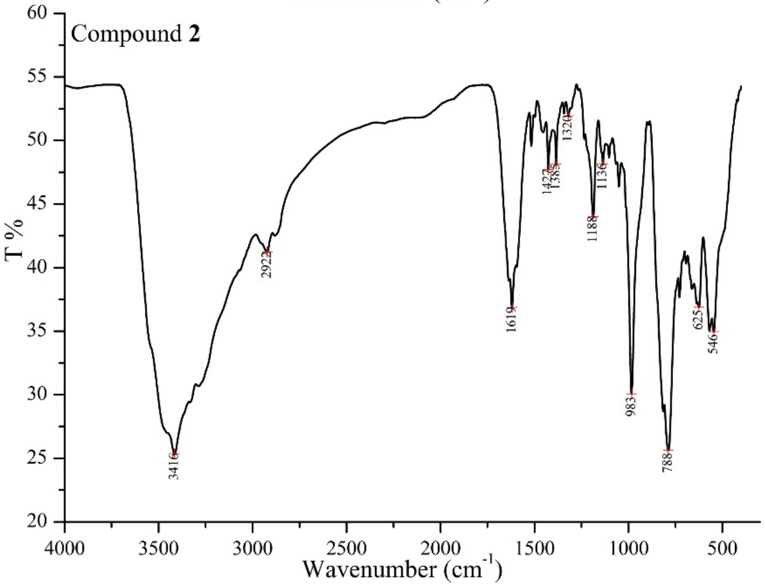
Compound 2
T %
Wavenumber (cm-1)
3416
2922
1619
1427
1385
1320
1188
1136
983
788
625
546

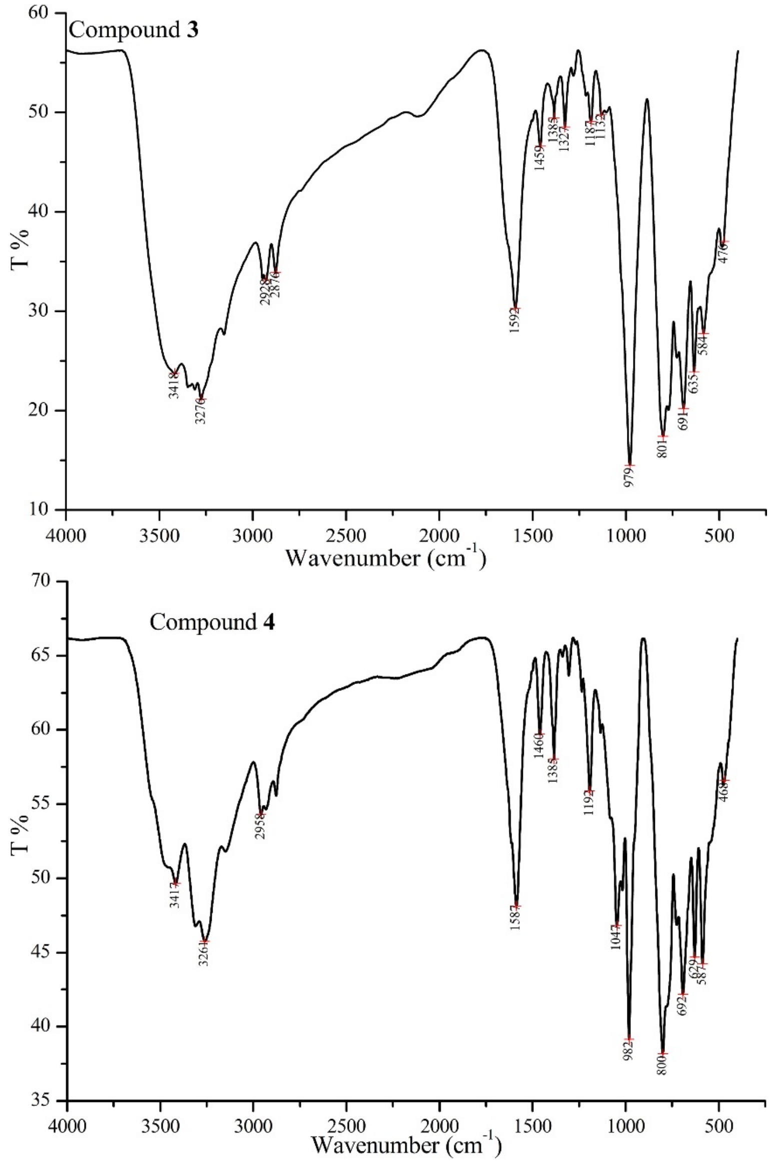

Figure S1. IR spectra of compounds **1**-**4**. The patterns of the bands in the region characteristic of $\nu(V{=}O_t)$ indicate the presence of V^{IV} sites: clusters which contain exclusively V^{IV} generally possess $\nu(V{=}O_t)$ bands in the range of 970-1000cm^{-1}, while bands in the region 940-960cm^{-1} are characteristic of V^{V}. The observation of a strong absorbance in the 970-1000cm^{-1} region provides a useful diagnostic for the presence of V^{4+} centers.[2] All the four compounds exhibits strong peaks around 980cm^{-1}, confirming the V^{4+} centres in all the four compounds.

C. Powder X-ray diffraction patterns spectra

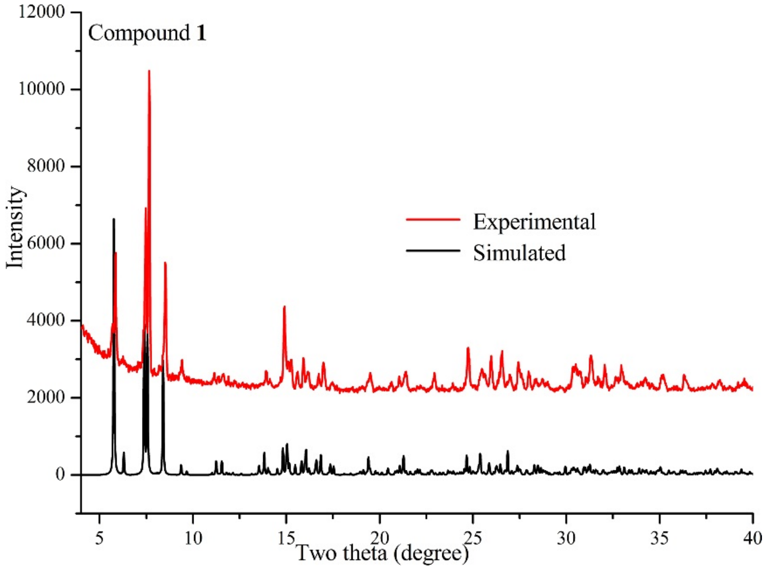

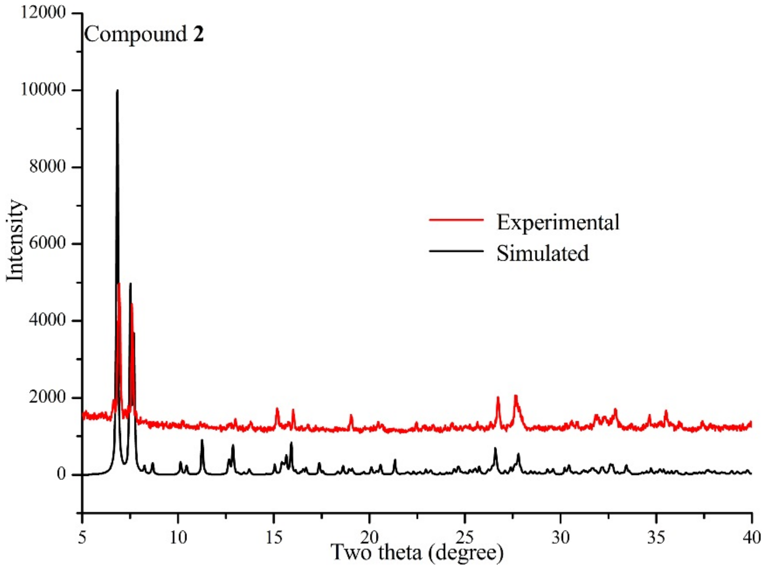

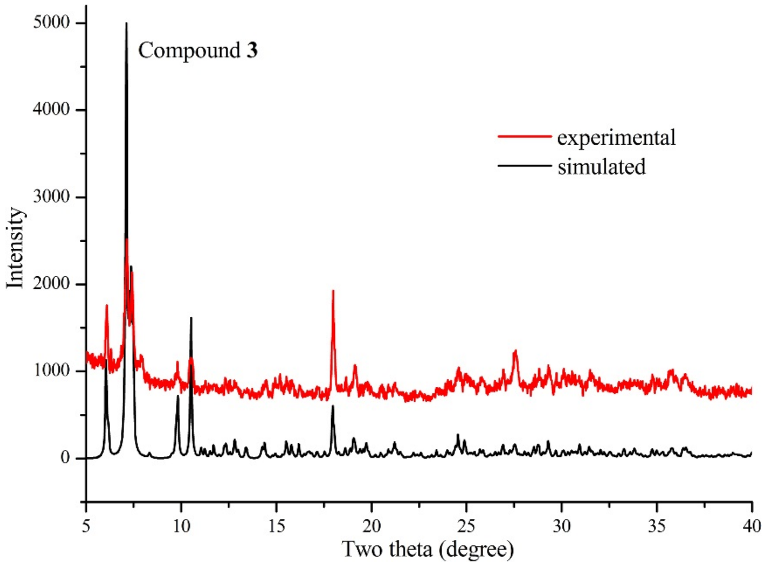

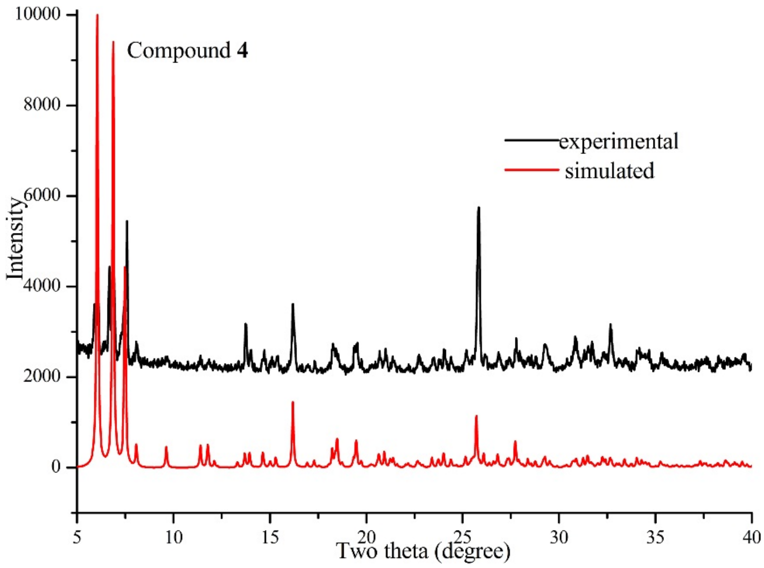

Figure S2. Simulated and experimental XRD patterns of compounds **1**-**4**.

D. UV-vis spectra

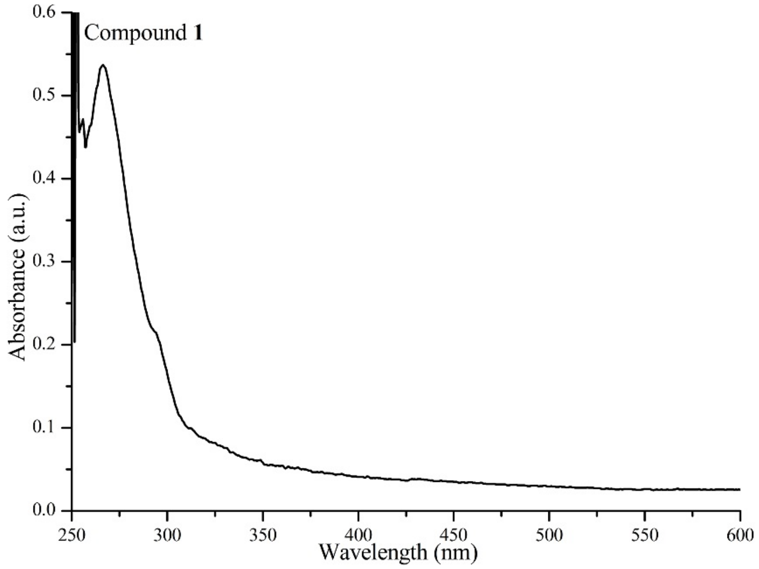

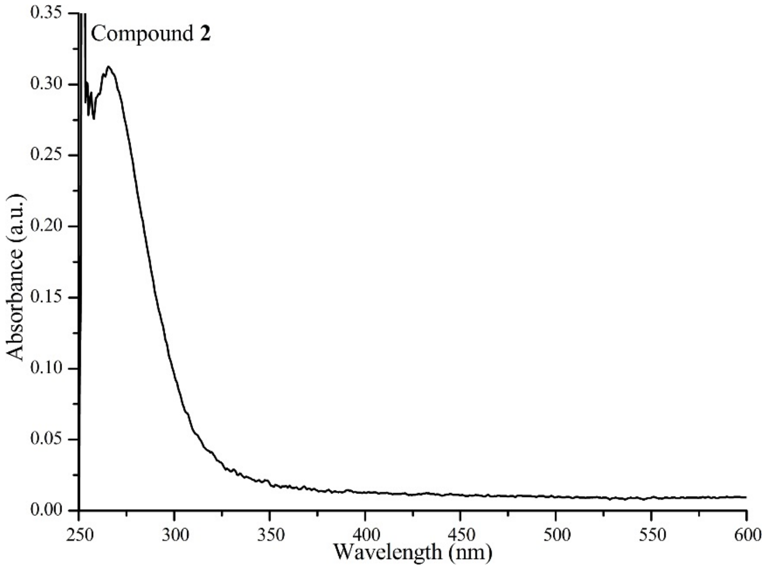

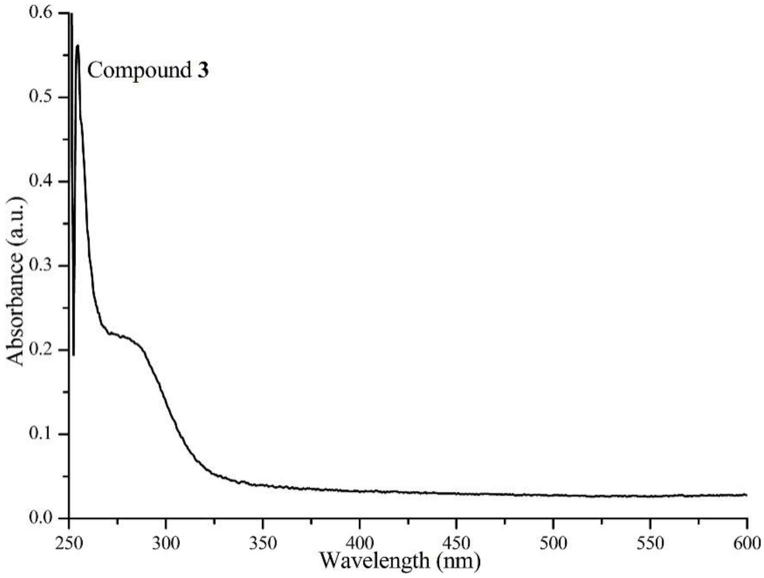

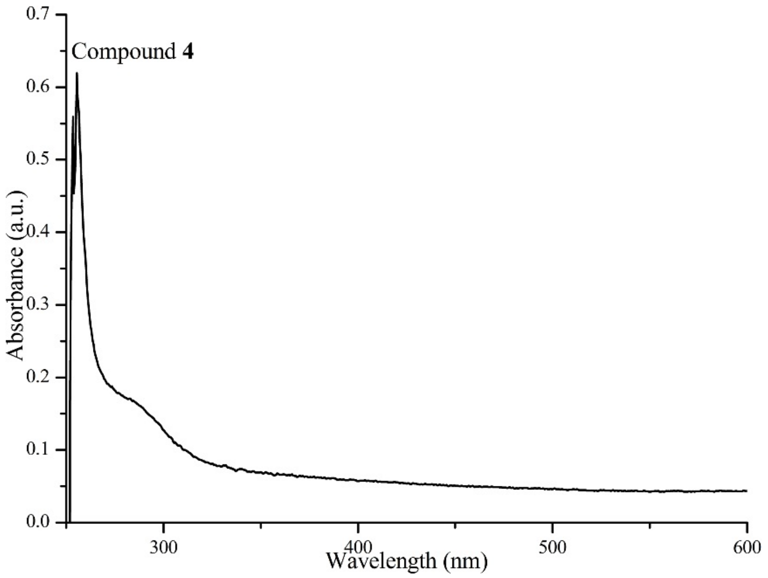

Figure S3. UV-Vis spectra of compounds **1**-**4**. There are no d-d transition bands in the UV-Vis spectra for all the three compounds, the reason is because the solution used is very dilute. Such phenomenon can be observed especially for the heterogenous supported catalysts. For the amount supported on the solids like Graphene oxide, SBA-15, molecule sieve and so on is very low, sometimes the vanadium d-d transitions cannot be observed. examples from our university: Z. F. Li, Appl. Organometal. Chem. 2012, 26, 252–257; Materials Research Bulletin, 48(2013) 1920.

E. ESR spectra

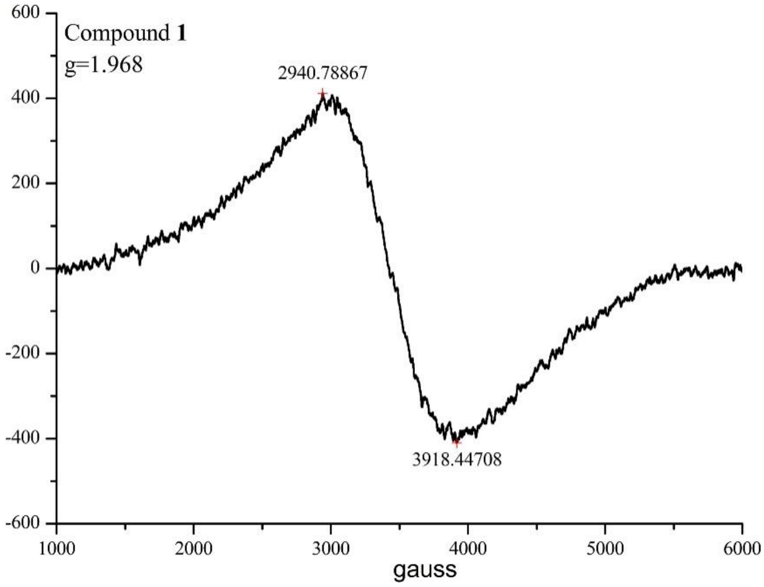

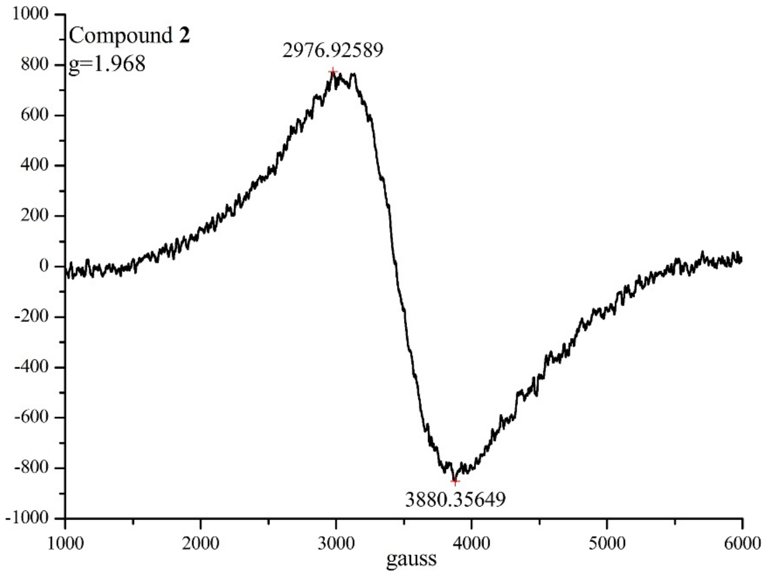

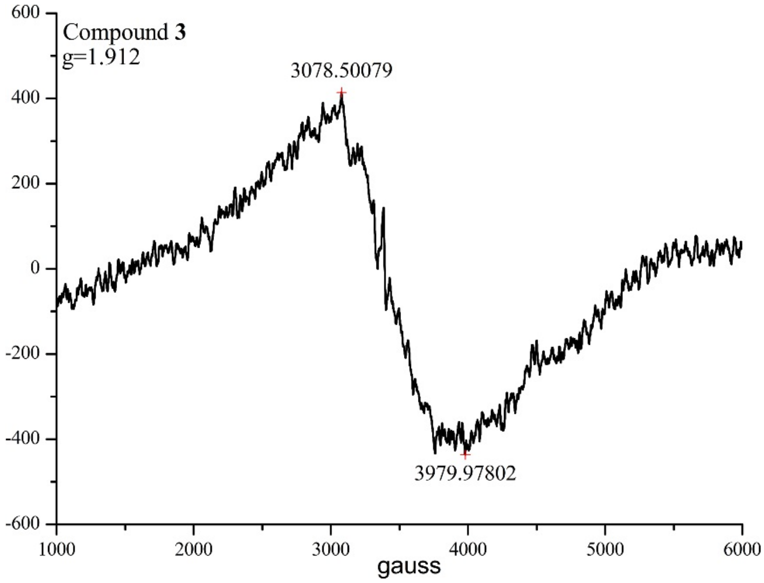

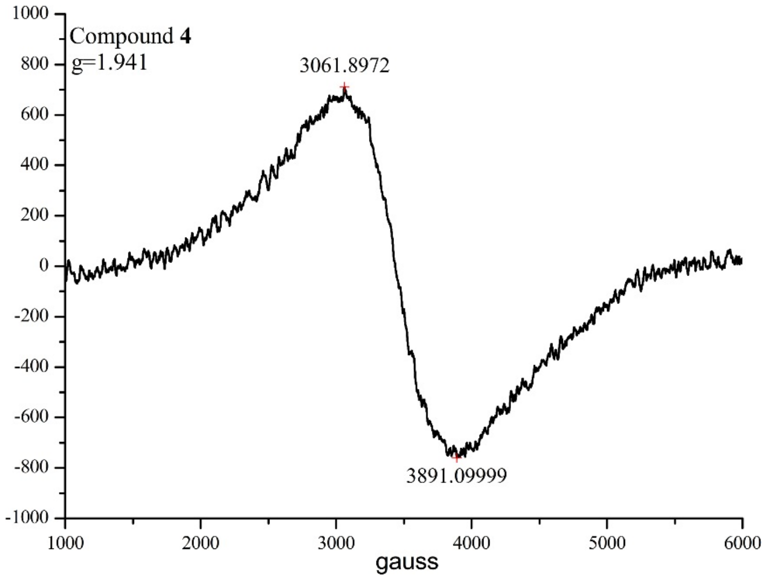

Figure S4. EPR spectra of compounds **1**-**4**. The ESR spectra of compounds **1**-**4** were studied at room temperature (Fig. S4.). The ESR spectra of compounds **1**-**4** are very similar to one another, which show Lorentzian shapes accompanied by signals at g = 1.968, 1.968, 1.912 and 1.941, respectively, indicating that the vanadium atoms in compounds **1**-**4** are in a +4-oxidation state. The ESR spectra further confirm the results of the bond valence sum calculations of compounds **1**-**4**.

F. The comparisons of the catalytic oxidation of styrene

Table S2. Comparison of the catalytic performances of our compounds and other reported POMs.

	Amount (mol)	Temp (ºC)	TBHP (mmol)	Time (h)	Conv. (%)	Epoxide selectivity (%)	Related work
$[Cu_4(3atrz)_4][PMo_{12}O_{40}]$	0.0041	80	3	10	92	11	[3]
$[Cu_6(3atrz)_6][PMo_{12}O_{40}]_2$	0.0041	80	3	10	91	8	[3]
$[Cu(bipy)]_4[Mo_{15}O_{47}]\cdot 2H_2O$	0.005	61	1	12	91	9.0	[4]
$CuI(bix)][(Cubix)(\delta\text{-}Mo_8O_{26})_{0.5}]$	0.005	61	1	12	70	11	[4]
Compound 1	0.0006	80	2	8	50.1	62.8	This work
Compound 2	0.0006	80	2	8	96.3	71.6	This work
Compound 3	0.0006	80	2	8	81.4	63.0	This work
Compound 4	0.0006	80	2	8	84.1	55.5	This work
Compound 5	0.0007	80	2	8	41.7	67.1	This work

G. X-ray diffraction patterns FT-IR spectra of compound 3 after the three cycles

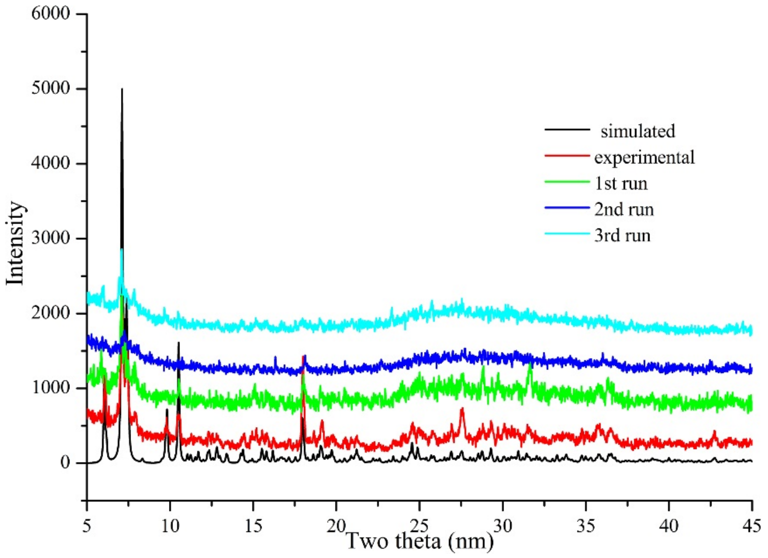

Figure S5. Simulated, experimental XRD patterns and XRD patterns after three cycles of compounds **3**.

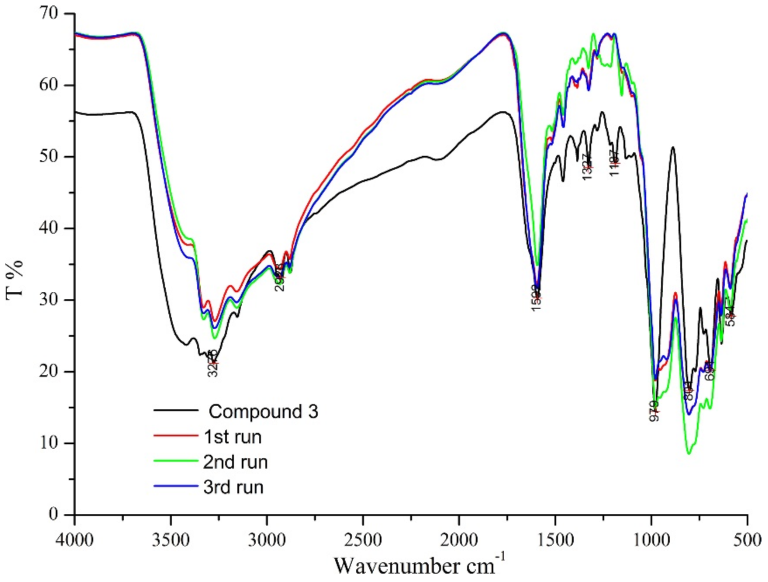

Figure S6. FT-IR spectrum of compound **3** and FT-IR spectra of compound **3** after three cycles.

H. References

References:

1. Brown, I.D.; Altermatt, D. Bond-valence parameters obtained from a systematic analysis of the Inorganic Crystal Structure Database. *Acta. Cryst.*, **1985**, *B41,* 244-247.
2. Keene, T.D.; D'Alessandro, D.M.; Krämer, K.W.; Price, J.R.; Price, D.J.; Decurtins, S.; Kepert, C.J. $[V_{16}O_{38}(CN)]^{9-}$: A Soluble Mixed-Valence Redox-Active Building Block with Strong Antiferromagnetic Coupling. *Inorg. Chem.*, **2012**, *51*, 9192-9199.
3. Gao, H.C.; Yan, Y.; Xu, X.H.; Yu, J.H.; Niu, H.L.; Gao, W.X.; Zhang, W.X.; Jia, M.J. Kinetics and mechanism of thymine degradation by TiO_2 photocatalysis. *Chin. J. Cata.*, **2015**, *36*, 1811-1824.

4. Song, X.J.; Yan, Y.; Wang, Y.N.; Hu, D.W.; Xiao, L.N.; Yu, J.H.; Zhang, W.X.; Jia, M.J. Hybrid compounds assembled from copper-triazole complexes and phosphomolybdic acid as advanced catalysts for the oxidation of olefins with oxygen. *Dalton Trans.*, **2017**, *46,* 16655-16662.
